# c-MYC is an aggregation-prone, amyloidogenic protein

**DOI:** 10.64898/2026.03.12.711438

**Published:** 2026-03-13

**Authors:** Ling Lin, Kun-Han Chuang, Chengkai Dai

**Affiliations:** 1 Mouse Cancer Genetics Program, Center for Cancer Research, National Cancer Institute, Frederick, MD 21702, USA; 2 Current address: School of Basic Medical Sciences, Fujian Medical University, Fuzhou, Fujian 350108, China; 6 Lead contact

## Abstract

As a master transcription factor, c-MYC governs a plethora of biological processes. Despite being a prominent oncoprotein, counterintuitively, c-MYC also possesses an intrinsic tumor suppressor activity through induction of apoptosis, a phenomenon known as “the paradox of c-MYC”. Serendipitously, we discover that c-MYC is aggregation-prone, becoming detergent-insoluble upon heat shock. Even in the absence of heat shock, c-MYC assembles into soluble oligomers exhibiting characteristics of amyloids in both human cancer tissues and, surprisingly, human Alzheimer’s disease brains. *In vitro*, recombinant c-MYC proteins form amyloid oligomers as well as protofibrils spontaneously. By contrast, its obligate dimerization partner MAX is non-amyloidogenic and, moreover, antagonizes the amyloidogenesis of c-MYC. Screening of the c-MYC synthetic peptide library identifies two intrinsically disordered short linear fragments, which are amyloidogenic *in vitro*. This amyloidogenesis of c-MYC, importantly, induces apoptosis largely independently of transcription. Thus, our findings unveil a previously unrecognized amyloidogenicity of c-MYC, which may contribute to its tumor-suppressing activity. Upon loss of the stringent control of its expression, conceptually, this amyloidogenesis of c-MYC may serve as a built-in failsafe mechanism to self-destruct its troublesome oncogenic potential.

## INTRODUCTION

The bHLH/ZIP transcription factor c-MYC plays vital roles in human biology, particularly acting as an oncogenic driver and a pluripotency-reprogramming factor.^1,2^ Through interaction with MYC-associated factor X (MAX), MYC/MAX dimers bind to the canonical E-box (5’-CACGTG-3’) element or its variants and regulate the transcription of 10–15% of the human genome.^1,3^ It is generally believed that dimerization with MAX is a prerequisite for the transcriptional activity of c-MYC.^4^ Unlike in primary, non-transformed cells, where its protein undergoes rapid turnover and remains at low levels, c-MYC expression is invariably elevated in human cancers, due to gene amplification, translocation, or protein stabilization.^5^ Despite being a prominent oncoprotein, paradoxically, c-MYC can elicit apoptosis, an intrinsic tumor-suppressing activity, especially under stressed conditions or with abundant expression.^6,7^ This induction of apoptosis, unsurprisingly, has been ascribed to its transcriptional action.^8,9^

Proteins including c-MYC naturally remain functional at the soluble and folded state; however, proteotoxic stressors and genetic mutations can cause protein misfolding and aggregation, leading to loss of function and cytotoxicity. Amyloids, a rare form of aggregates enriched with cross-β-sheet structures, have been closely implicated in human diseases particularly neurodegenerative disorders.^10^ Two fluorescence dyes, Congo red (CR) and thioflavin T (ThT), have been extensively applied to detect amyloids through physical binding.^11^ In addition, the conformation-specific antibody A11 has become an invaluable tool for recognizing the soluble prefibrillar amyloid oligomers (AOs), a conformer highly toxic to neurons, independently of primary amino acid sequences.^12^ Beyond detection, CR has been known to impede the amyloidogenic process.^13,14^

Serendipitously, our studies now discover that c-MYC, but not its obligate dimerization partner MAX, proteins become detergent-insoluble aggregates upon heat shock (HS). Furthermore, in human cancer tissues and, surprisingly, Alzheimer’s disease (AD) brains c-MYC assembles into detergent-soluble oligomers captured by the A11 antibody even in the absence of HS. Interestingly, this aggregation and oligomerization can be blocked by CR. *In vitro*, recombinant c-MYC, but not MAX, proteins spontaneously form AOs as well as protofibrils, demonstrating the intrinsic amyloidogenicity of c-MYC. MAX, intriguingly, prevents the amyloidogenesis of c-MYC. By screening a synthetic peptide library of human c-MYC, two intrinsically disordered short linear fragments (aa20–39 and aa210–228) are identified to be amyloidogenic. Moreover, a large majority of amino acids within these two fragments contribute to their amyloidogenicity, revealed by alanine scan. Unexpectedly, the transcription-deficient c-MYC mutant, which lacks the C-terminal MAX-dimerizing and DNA-binding domain, can still induce apoptosis. Deletion of these two amyloidogenic regions, importantly, largely blocks this apoptosis. Taken together, our data pinpoint c-MYC as an aggregation-prone, amyloidogenic protein. Biologically, the amyloid state of c-MYC prompts apoptosis independently of transcription, fully unleashing its tumor-suppressing potential. Thus, our findings both provide new insights into the paradox of c-MYC and uncover its complex implications in human pathologies, including cancer and neurodegeneration.

## RESULTS

### c-MYC acquires amyloid-like attributes under stressed and pathological conditions

We recently discovered that under non-stressed conditions heat shock factor 1 (HSF1) potentiates the c-MYC-mediated transcription via physical interactions, a non-canonical transcriptional action of HSF1.^15^ Subsequently, we were curious about how this c-MYC-HSF1 interaction is affected by proteotoxic stress, such as HS, wherein HSF1 is obligatory for initiating the canonical heat-shock, or proteotoxic stress, response (HSR/PSR).^16,17^

**Figure 1:**
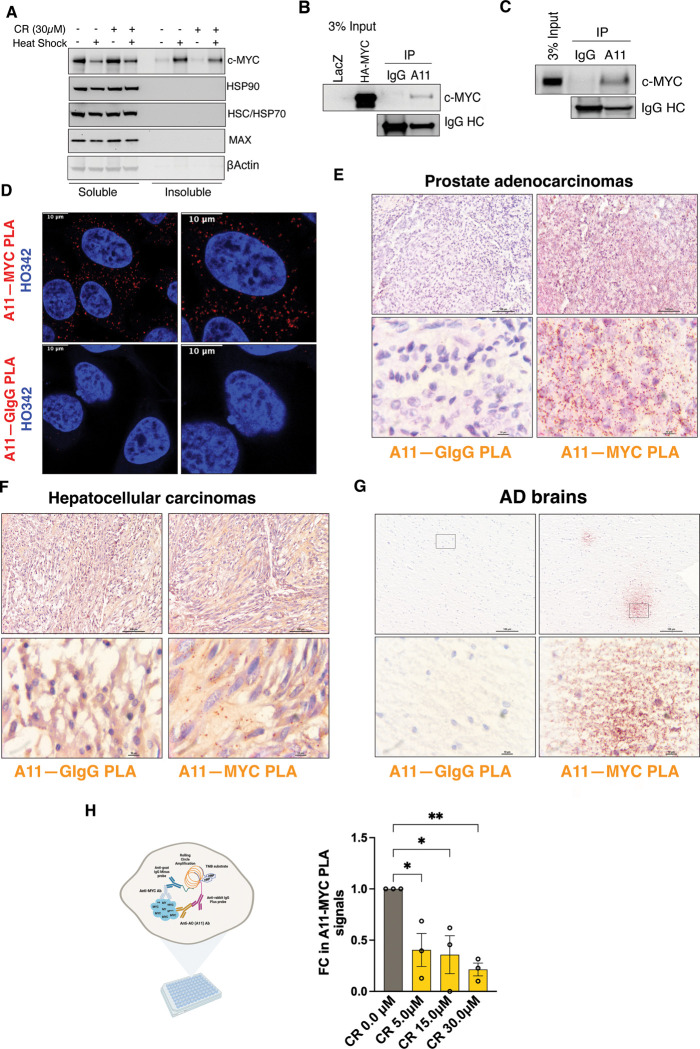
c-MYC becomes detergent-insoluble and displays amyloid-like properties under proteotoxic stress and pathological conditions. (A) Detection of detergent-soluble and - insoluble c-MYC proteins by immunoblotting in HeLa cells pre-treated with 30 μM CR, followed by HS at 43°C for 30 min (representative images of three independent experiments). (B) and (C) Immunoprecipitation of either exogenously expressed HA-c-MYC or endogenous c-MYC proteins in HeLa cells by A11 antibodies (representative images of three independent experiments). (D) Detection of endogenous c-MYC proteins in HeLa cells by PLA using both the goat anti-c-MYC Ab and the rabbit anti-AO (A11) Ab. Nuclei are counterstained with Hoechst 33342. Scale bar: 10 μm. (E)-(G) Detection of endogenous c-MYC proteins in human prostate adenocarcinoma, hepatocellular carcinoma, and Alzheimer’s disease brain tissues by brightfield PLA using both the goat anti-c-MYC Ab and the rabbit anti-AO (A11) Ab. Scale bar: 100 μm for low magnification and 10 μm for high magnification. (H): Quantitation of endogenous c-MYC recognized by both the goat anti-c-MYC Ab and the rabbit anti-AO (A11) Ab through In-Cell PLA ELISA in HeLa cells treated with CR (mean ± SD, n=3 independent experiments, One-way ANOVA).

In HeLa cells transient HS, unexpectedly, caused a notable reduction in detergent-soluble c-MYC proteins, accompanied with an increase in detergent-insoluble proteins (Figure 1A). This transition in physical states suggests c-MYC aggregation upon proteotoxic stress. By sharp contrast, its obligate dimerization partner MAX did not aggregate. Neither did major cellular chaperones including HSP90 and HSC/HSP70 (Figure 1A), which are much more abundant than c-MYC inside cells. Of note, this c-MYC aggregation was partially blocked by CR (Figure 1A). Despite being a popular amyloid stain known to impede amyloidogenesis, CR may also counteract generic protein aggregation. To determine whether c-MYC could form amyloid-like aggregates, we took advantage of the conformation-specific A11 antibody, which has been widely applied to recognize soluble prefibrillar AOs.^12^ In HeLa cells, A11 antibodies precipitated both exogenous HA-c-MYC and endogenous c-MYC proteins in the absence of HS (Figures 1B and 1C). This finding was further confirmed by *in situ* Proximity Ligation Assay (PLA), ^18^ a technique detecting protein-protein interactions or two distinct epitopes located on the very same protein, in cultured HeLa cells (Figure 1D). The PLA signals were quantitated by flow cytometry (Figure S1A). Of note, these PLA signals were largely localized in the cytoplasm (Figure 1D), suggesting that these special c-MYC conformers are incompetent at transcription. The emergence of such conformers is not due to cell culture artifacts, as these A11^+^-c-MYC oligomers were readily detected in human cancer tissues, both primary and xenografted (Figures 1E, 1F, and S1B). Intriguingly, such c-MYC oligomers were also detected in human AD brain tissues (Figure 1G), resembling diffuse Aβ plaques. Importantly, the genesis of A11^+^-c-MYC oligomers was blocked by CR treatment (Figures 1H and S1C), further corroborating the amyloidogenic nature of such aggregates. Together, these findings support that c-MYC proteins, at least under proteotoxic stress and certain pathological conditions, could demonstrate some characteristics of amyloids.

### c-MYC is intrinsically amyloidogenic

It is worth pointing out that both the immunoprecipitation and PLA staining only indicate that either c-MYC interacted with AOs or c-MYC itself became amyloids. To distinguish these two possibilities, we next resorted to *in vitro* assays using purified recombinant proteins.

**Figure 2:**
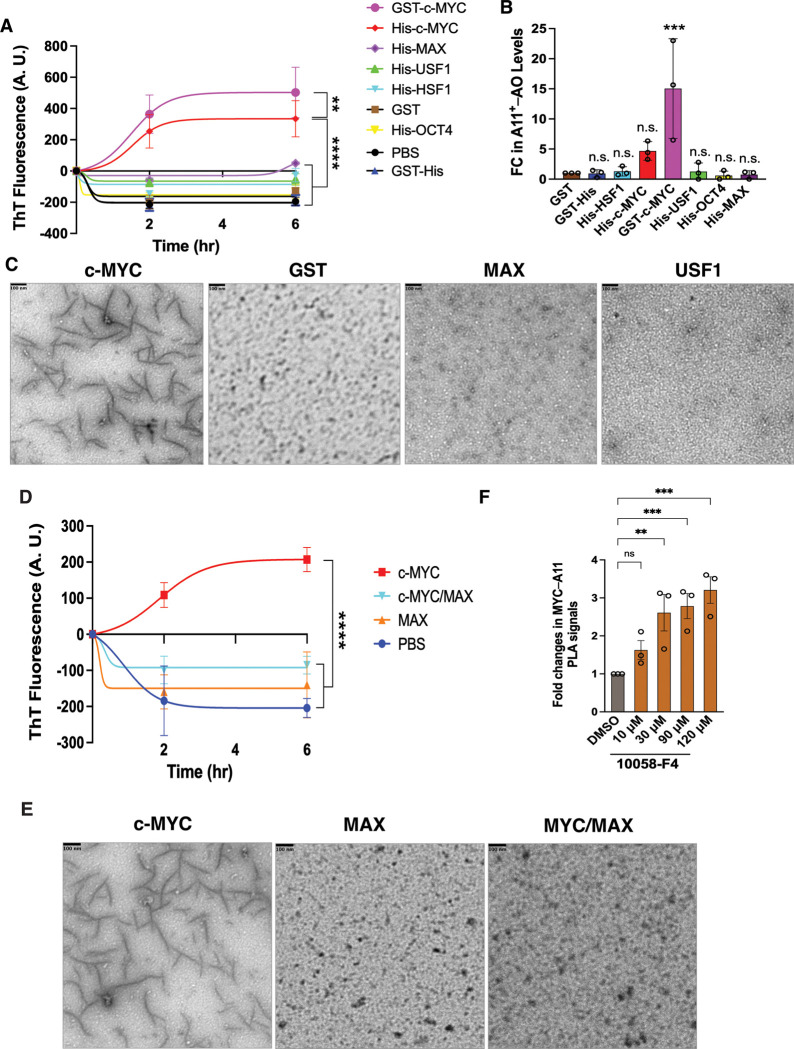
c-MYC is intrinsically amyloidogenic. (A) *In vitro* fibrillation assays using recombinant c-MYC proteins (mean ± SEM, n=3 independent experiments, Two-way ANOVA). (B) ELISA quantitation of A11^+^-AOs generated by recombinant c-MYC proteins *in vitro* (mean ± SD, n=3 independent experiments, One-way ANOVA). (C) TEM visualization of protofibrils formed by recombinant c-MYC proteins *in vitro* (representative images of three independent experiments). Scale bars: 100 nm. (D) *In vitro* fibrillation assays using recombinant c-MYC /MAX dimers (mean ± SEM, n=3 independent experiments, Two-way ANOVA). (E) TEM visualization of recombinant c-MYC/MAX dimers *in vitro* (representative images of three independent experiments). Scale bars: 100 nm. (F) Quantitation of endogenous c-MYC AOs in HeLa cells treated with 10058-F4 by In-Cell PLA ELISA (mean ± SD, n=3 independent experiments, One-way ANOVA).

In the classic *in vitro* fibrillation assay, recombinant c-MYC proteins, prepared by two independent sources, spontaneously assembled into amyloid fibrils, detected by ThT binding (Figure 2A). This sharply contrasted with GST and a panel of recombinant transcription factors including HSF1, USF1, OCT4, as well as MAX (Figure 2A). Of note, neither MAX, the obligate dimerization partner of c-MYC, nor USF1, another member of the c-MYC family, formed amyloid fibrils, underlining the specific amyloidogenicity of c-MYC. Aside from amyloid fibrils, recombinant c-MYC proteins also assembled into soluble AOs, recognized by the A11 antibody (Figure 2B). Likely due to the solubilizing effect of GST tags, GST-c-MYC proteins produced markedly more AOs. Transmission electron microscopy (TEM) further confirmed the assembly of protofibrils from recombinant c-MYC proteins (Figure 2C). Collectively, these *in vitro* studies indicate that c-MYC itself is an amyloidogenic protein.

Considering that c-MYC dimerizes with non-amyloidogenic MAX under physiological conditions,^4^ we were intrigued by the question of how this dimerization impacts the amyloidogenesis of c-MYC. First, the *in vitro* fibrillation assay revealed that recombinant c-MYC/MAX heterodimers did not form amyloid fibrils, in stark contrast to recombinant c-MYC proteins alone (Figures 2D and 2E). Second, disruption of the MYC/MAX dimerization in HeLa cells using the small molecule 10058-F4 led to increased A11^+^–c-MYC oligomers, despite reduced soluble c-MYC proteins (Figures 2F, S2A, and S2B).^19^ These findings together strongly support that dimerization with MAX suppresses the amyloidogenesis of c-MYC.

### Deciphering the intrinsic amyloidogenicity of c-MYC

**Figure 3:**
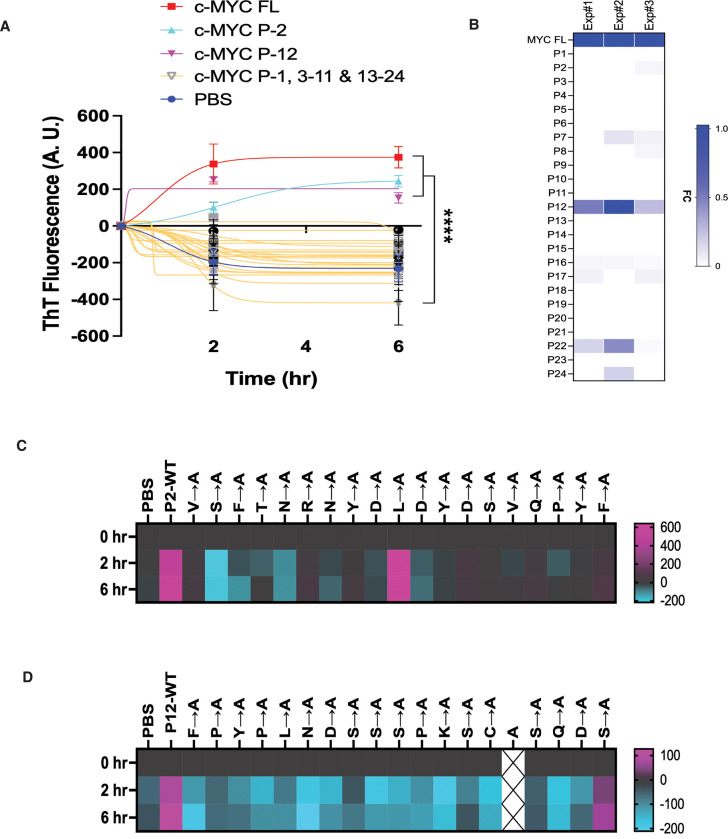
Decoding the amyloidogenicity of c-MYC *in vitro*. (A) *In vitro* fibrillation assays using the human c-MYC peptide library (mean ± SEM, n=3 independent experiments, Two-way ANOVA). (B) ELISA quantitation of A11^+^-AOs generated by synthetic c-MYC peptides. Results of three independent experiments are presented as a heatmap. FC: fold change. (C) and (D) *In vitro* fibrillation assays using the P2 and P12 peptides that are subjected to alanine scan. Results are presented as heatmaps using the averages of three independent experiments.

To systematically dissect the amyloidogenic regions, we designed a synthetic peptide library of human c-MYC proteins, each peptide encompassing 19 non-overlapping amino acids. This technique has been successfully applied to delineate protein-protein interaction interfaces previously.^15^ Among total 24 linear peptides, only two, P2 (aa20–38) and P12 (aa210–228), displayed evident amyloidogenesis, like the full-length c-MYC protein, indicated by the *in vitro* fibrillation assay (Figure 3A). Of interest, only P12 consistently assembled into A11^+^-AOs as well (Figure 3B). It is noteworthy that both P2 and P12 are located within the intrinsically disordered regions (IDRs) of c-MYC (Figure S3A).

To further pinpoint individual amino acids critical to this amyloidogenicity, both P2 and P12 were subjected to alanine scan. Revealed by *in vitro* fibrillation assays, in most cases mutation of individual amino acids markedly diminished amyloid fibrillation (Figures 3C and 3D). These findings indicate that intrinsically disordered short linear fragments can serve as the amyloidogenic regions. Furthermore, it appears that numerous residues within these regions collectively contribute to the amyloidogenicity.

### Illustrating the biological implications of c-MYC amyloidogenesis

**Figure 4:**
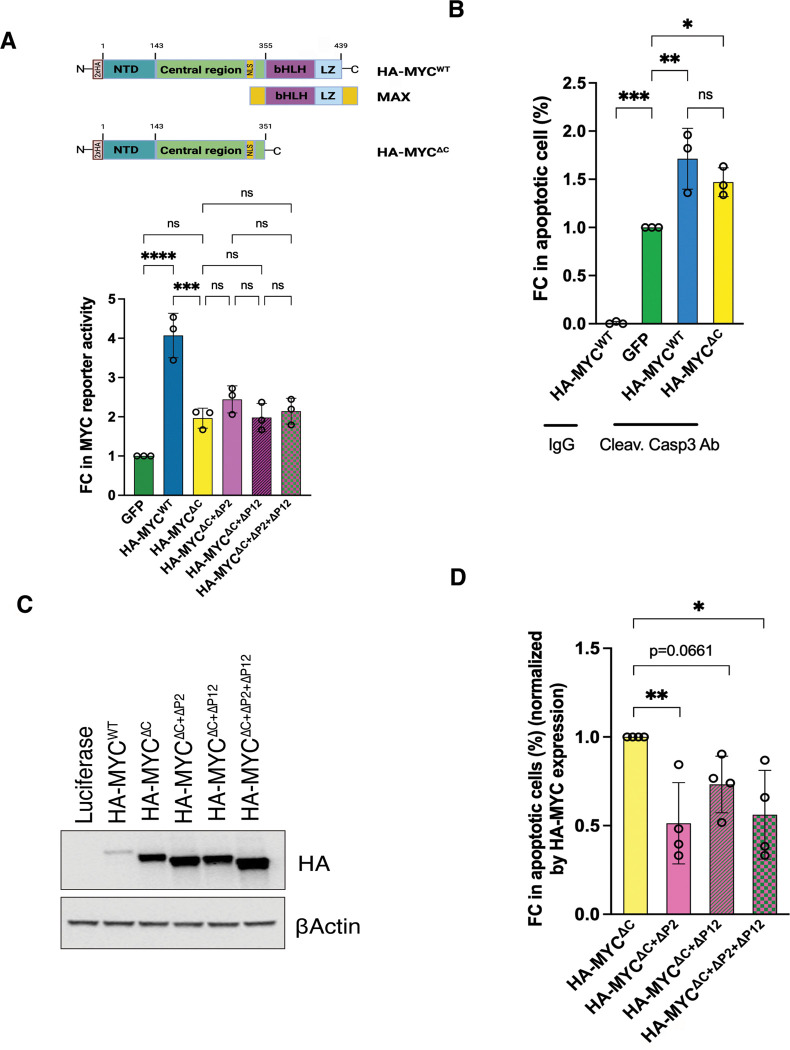
The amyloidogenesis of c-MYC contributes to its intrinsic tumor suppressor activity. (A) Measurements of the transcriptional activities of HA-MYC^WT^ and HA-MYC^ΔC^ in HeLa cells using the dual MYC reporter system (mean ± SD, n=3 independent experiments, One-way ANOVA). (B) Quantitation of apoptosis induced by transient expression of HA-MYC^WT^ or HA-MYC^ΔC^ in serum-starved NIH3T3 cells by flow cytometry using anti-cleaved caspase 3 Abs (mean ± SD, n=3 independent experiments, One-way ANOVA). (C) Detection of HA-MYC expression in serum-starved NIH3T3 cells by immunoblotting (representative images of four independent experiments). (E) Quantitation of apoptosis induced by transient expression of HA-MYC^ΔC^ and its deletion mutants in serum-starved NIH3T3 cells by flow cytometry using anti-cleaved caspase 3 Abs. The results are normalized by their expression levels (mean ± SD, n=4 independent experiments, One-way ANOVA).

Aggregates, especially amyloids, are cytotoxic. We reasoned that the amyloidogenesis of c-MYC might cause apoptosis, contributing to its tumor-suppressing effect. It has been well documented that c-MYC can trigger apoptosis under stressed conditions, including serum starvation and DNA damage, or simply with elevated expression. ^6–9^ To distinguish the transcription-induced from amyloid-induced apoptosis, we generate the transcription-deficient HA-c-MYC^ΔC^ mutant, which lacks the C-terminal DNA-binding and MAX-dimerizing domains but retains the amyloidogenic regions (Figure 4A). Overexpression of HA-c-MYC^WT^, as expected, induced apoptosis in serum-starved NIH-3T3 cells, detected by caspase 3 cleavage (Figures 4B). So did HA-c-MYC^ΔC^ mutants (Figures 4B), suggesting a surprising transcription-independent mechanism. Naturally, we asked whether the amyloidogenesis of HA-c-MYC^ΔC^ contributes to this apoptosis. To this end, the two amyloidogenic regions were deleted from HA-c-MYC^ΔC^. Of note, deletions of these regions altered the expression of HA-c-MYC^ΔC^ (Figure 4C). The apoptosis-inducing effects were normalized by their expression levels accordingly. While deletion of the P2 region markedly diminished apoptosis, deletion of the P12 region was less effective (Figure 4D). Of note, deletion of the amyloidogenic regions did not further impair the residual transcriptional activity of HA-c-MYC^ΔC^ mutants (Figure 4A); by contrast, it did significantly diminish the apoptosis-inducing capability of these mutants (Figure 4D), decoupling apoptosis induction from transcriptional activity. In aggregate, these findings strongly support that the amyloidogenesis of c-MYC provokes cytotoxicity, which could contribute to its intrinsic tumor suppressor activity.

## DISCUSSION

### c-MYC is an aggregation-prone and amyloidogenic protein

Our combined *ex vivo* and *in vitro* studies provide compelling evidence pinpointing c-MYC as an aggregation-prone and amyloidogenic protein. This conclusion is drawn from multiple independent lines of evidence, including the blockade of c-MYC amyloidogenesis by CR, ThT binding of c-MYC amyloid fibrils, recognition of c-MYC AOs by A11 antibodies, as well as direct visualization of c-MYC amyloid protofibrils by TEM. Most importantly, we have delineated two intrinsically disordered short linear fragments as the amyloidogenic regions. Although both short fragments assembled into amyloid fibrils *in vitro*, indicated by ThT binding, only one of them, P12, consistently produced A11^+^–AOs. It remains possible that P2 may assemble into a distinct class of AOs, which cannot be recognized by the A11 antibody.

### MAX acts as a suppressor of c-MYC amyloidogenesis

One intriguing discovery is that its obligate dimerization partner MAX, which is non-amyloidogenic, antagonizes the amyloidogenesis of c-MYC. This finding predicts that in primary, non-transformed cells, where c-MYC levels remain low and sufficient MAX is available for dimerization, the amyloidogenesis of c-MYC would be curtailed. However, a vastly different scenario would be expected in cancerous cells, where c-MYC levels are markedly elevated and, importantly, the expression of *c-MYC* and *MAX* are poorly correlated (Figures S4A, S4B, and S4C). Conceivably, c-MYC is selected for overexpression owing to its oncogenic potential; by contrast, MAX is not subjected to the same selection pressure, owing to its tumor-suppressing effects through dimerization with MXD family proteins. ^20,21^ This disproportionate c-MYC and MAX expression, likely, unleashes the amyloidogenesis of c-MYC in human cancers.

### New insights into the paradox of c-MYC

“The paradox of c-MYC” depicts an intriguing dichotomy, in which c-MYC acts as an oncoprotein with intrinsic tumor suppressor activity. This stimulation of apoptosis, traditionally, has been exclusively ascribed to the transcriptional action of c-MYC. Our studies now reveal that the amyloid state of c-MYC provokes apoptosis in a largely transcription-independent fashion. Thus, c-MYC can exert its tumor suppressor activity via both transcription-dependent and -independent mechanisms, likely contingent upon the cellular c-MYC abundance. Given its fundamental roles in biology, unsurprisingly, the expression of c-MYC is subjected to exceedingly tight control under physiological conditions, loss of which would inevitably lead to pathogeneses particularly malignancy. Conceptually, this amyloidogenic process could serve as a built-in failsafe mechanism for c-MYC to self-destruct its troublesome oncogenic potential.

### Implications of c-MYC amyloidogenesis in cancer and neurodegeneration

Previously, we uncovered the phenomenon of “tumor-suppressive amyloidogenesis”, ^14^ owing to widespread proteomic instability of cancer. Now, our findings further pinpoint c-MYC as an exemplar of tumor-associated amyloids. Given their cytotoxic impact, these amyloid conformers of c-MYC must be contained to avert their tumor-suppressing effects in cancerous cells. Our previous study revealed that HSF1, the master regulator of the HSR/PSR, could physically neutralize toxic AOs. ^22^ Intriguingly, HSF1 happens to interact with c-MYC. ^15^ Thus, in cancerous cells HSF1 may help counteract these c-MYC amyloids. In accordance with this notion, the expression of *HSF1* and *c-MYC* is well correlated in human cancers.^15^

Beyond cancer, our data unveil that the amyloidogenesis of c-MYC manifests in human AD as well. In fact, it has been reported that MYC expression is elevated in human AD brains.^23,24^ Moreover, overexpression of *c-MYC* in neurons led to neurodegeneration in mice, an effect attributed to neuronal cell cycle re-entry. ^25^ Our findings, nevertheless, suggest an implication of c-MYC amyloidogenesis in neurodegenerative disorders, a notion warranting further investigations.

## EXPERIMENTAL METHODS

### Cell Lines and Reagents

HeLa (female) cells and NIH3T3 (male) cells were purchased from ATCC. They were authenticated by ATCC by STR profiling. All cell cultures were maintained in DMEM supplemented with 10% HyClone bovine growth serum and 1% penicillin–streptomycin. Cells were maintained in an incubator with 5% CO2 at 37 °C. All cell lines were routinely tested for mycoplasma contamination using MycoAlert Mycoplasm Detection kits.

Recombinant proteins were all purchased commercially, including c-MYC/MAX complexes (cat#81087, Active Motif Inc.), His-HSF1 (cat#ADI-SPP-900-F, Enzo Life Sciences Inc.), GST-c-MYC (cat#M86–30G, Sino Biological US Inc.), His-c-MYC (cat#230–00580-100, RayBiotech Inc.), GST (cat#G52–30U-50, SignalChem Inc.), His-GST (cat#RPT0002, ABclonal Inc.), His-MAX (cat#81017, Active Motif Inc.), His-USF1 (cat#228–21891-2, RayBiotech Inc.), and His-OCT4 (cat#228–21139-2, RayBiotech Inc.).

All antibodies were purchased commercially, including rabbit polyclonal anti-amyloid oligomer (A11) Ab (cat#SPC-506, StressMarq Biosciences Inc.), rabbit monoclonal anti-c-MYC/N-MYC (D3N8F) Ab (cat#13987, Cell Signaling Technology), rabbit monoclonal ani-MAX (E6F6Y) Ab (cat#17471, Cell Signaling Technology), mouse monoclonal anti-HSC/HSP70 (W27) Ab (cat#sc-24, Santa Cruz Biotechnology Inc.), mouse monoclonal anti-HSP90α/β (F-8) Ab (cat#sc-13119, Santa Cruz Biotechnology Inc.), rabbit monoclonal anti-cleaved caspase 3 (Asp175) (D3E9) Ab (cat#9579, Cell Signaling Technology), goat polyclonal anti-c-MYC Ab (cat#AF3696, R&D Systems Inc.), mouse monoclonal anti-HA Ab (cat#901513, BioLegend Inc.), mouse monoclonal anti-βActin (GT5512) Ab (cat#GTX629630, GeneTex Inc.), normal rabbit IgG (cat#02–6102,ThermoFisher Scientific Inc.), Peroxidase AffiniPure Goat Anti-Rabbit IgG (H+L) (cat#111–035-144, Jackson ImmunoResearch Inc.), Peroxidase AffiniPure Goat Anti-Mouse IgG (H+L) (cat#115–035-003, Jackson ImmunoResearch Inc.), and Peroxidase IgG Fraction Monoclonal Mouse Anti-Goat IgG, light chain specific (cat#205–032-176, Jackson ImmunoResearch Inc.).

All chemicals were purchased commercially, including Thioflavin T (ThT) (cat# AC211760050, ThermoFisher Scientific Inc.), Congo red (CR) (cat#C580–25, ThermoFisher Scientific Inc.), Janus Green B (cat# AC191680050, Fisher Scientific LLC), and 10058-F4 (cat#T3048, TargetMol Chemicals Inc.).

pcDNA-GFP and pcDNA3–2xHA-MYC were gifts from Martine Roussel (Addgene plasmid#74165 and 74164, respectively). pcDNA3–2xHA-MYC^ΔC^, 2xHA-MYC^ΔC+ΔP2^, 2xHA-MYC^ΔC+ΔP12^, and 2xHA-MYC^ΔC+ΔP2+ΔP12^ were generated using the Q5 Site-Directed Mutagenesis kit (cat#E0554S, New England Biolabs Inc.). pMYC-SEAP was purchased from Clontech Laboratories (Cell signaling pathway profiling systems, cat#631910) and pCMV-Gaussia Luc was purchased from Thermo Fisher Scientific (cat#16147).

The c-MYC peptide library and alanine scan libraries were custom synthesized by GenScript USA Inc.

Paraffin sections of human liver cancer (cat#HuCAT081) and prostate cancer (cat#HuCAT371) were purchased from TissueArray.com LLC. Paraffin sections of human Alzheimer’s disease brains (cat#CSA0225P) were purchased from American MasterTech Scientific. Paraffin sections of human A2058 melanomas were prepared from xenografted tumors described previously. ^14^

### Soluble and Insoluble Fractionation

HeLa cells were treated with Congo red for 24 hours, followed by incubation at 43°C for 30 min. Cells were then trypsinized, washed, and resuspended in 1xPBS for cell counting. Equal numbers of cells were used for soluble and insoluble fractionation. The detailed procedures were described previously. ^22^

### In-Cell PLA ELISA

To avoid the interference of CR or 10058-F4 in PLA fluorescence detection, In-Cell PLA ELISA was adopted using the Duolink^®^ In Situ Detection Reagent Brightfield (cat#DUO92012, Sigma-Aldrich). The detailed procedures were described previously.^15^ HeLa cells were seeded in 96-well culture plates (7000 cells/well) and treated with CR for 24 hr or 10058-F4 for 30 hr. The goat anti-c-MYC antibody and the rabbit anti-AO (A11) antibody were combined for the PLA, followed by incubation with Duolink^®^ PLA anti-rabbit Plus (cat#DUO92002, Sigma-Aldrich) and anti-goat Minus probes (cat#DUO92006, Sigma-Aldrich). Of note, 1-Step Ultra TMB-ELISA Substrate Solution (cat#34029, ThermoFisher Scientific), instead of the precipitating substrate provided by the kit, was used for the substrate development.

### Immunoblotting and Immunoprecipitation

Whole-cell protein extracts were prepared in cold cell-lysis buffer (50mM Tris-Cl, pH 7.5, 120mM NaCl, 0.5% NP-40 and 1mM PMSF). Proteins were transferred to nitrocellulose membranes. Following incubation with the blocking buffer (5% non-fat milk in 1x TBS-T) for 1 hour at RT, membranes were incubated with primary antibodies (1:1,000 dilution in the blocking buffer) overnight at 4 °C. After washing with 1xTBS-T for 3 times, membranes were incubated with peroxidase-conjugated secondary antibodies (1: 5000 diluted in the blocking buffer) at RT for 1 hr. Signals were detected using SuperSignal West chemiluminescent substrates (cat#34580 or 34095, Thermo Fisher Scientific Inc.).

For the A11 IP, HeLa cells were seeded for 24 hours or transfected with plasmid DNAs for 24 hours. Following trypsinization and washing in ice-cold 1xPBS, cells were lysed in cell lysis buffer on ice for 30 min and the extracts were centrifuged at 16,813×g for 5 min at 4°C to remove insoluble fractions. Soluble cell lysates (5 mg) were incubated with either rabbit IgG or anti-amyloid oligomers (A11) antibody (5 μg) at 4°C with gentle rotation for overnight. The immune complexes were then recovered with Dynabeads^™^ Protein G (cat#10004D, Life Technologies Corp.) or ChIP-Grade Protein G agarose beads (cat#9007S, Cell Signaling Technology). After washing with the lysis buffer, precipitates were subjected to SDS-PAGE and immunoblotting with either goat anti-c-MYC Abs or mouse anti-HA Abs, followed by incubation with HRP-conjugated bovine anti-goat IgG (cat#805–035-180, Jackson ImmunoResearch Laboratories Inc.) or EasyBlot^®^ anti-mouse IgG (HRP) (cat#GTX221667–01, GeneTex Inc.). Signals were detected using SuperSignal^™^ West Pico PLUS chemiluminescent substrates and documented with an iBright FL1000 Imaging System (ThermoFisher Scientific).

### *In Situ* Proximity Ligation Assay (PLA)

The general procedures for *in situ* PLA were described previously.^15^ For the specific MYC-A11 *in situ* PLA, the goat anti-c-MYC antibody and the rabbit anti-AO (A11) antibody were used, followed by incubation with Duolink^®^ PLA anti-rabbit Plus and anti-goat Minus probes. Images were captured using a Zeiss LSM780 confocal microscope.

Brightfield *in situ* PLA was performed using the Duolink^®^ In Situ Detection Reagents Brightfield. Paraffin-embedded tissue sections were deparaffinized at 60°C for 1.5 hr and rehydrated. Antigen retrieval was then performed using sodium citrate buffer (pH 6.0) in a vegetable steamer for 25 min. Following permeabilization using 0.3% Triton-X-100 in 1xPBS at RT for 10 min, endogenous peroxidase and alkaline phosphatase activities were quenched using the BLOXALL blocking solution at RT for 10 min and slides were blocked with 1x Duolink blocking solution (cat#DUO82007, Sigma-Aldrich) at 37°C for 1 hr. The tissue slides were incubated with rabbit anti-AO (A11) Abs (1:100) and goat anti-c-MYC Ab (1:100) or goat normal IgG (Jackson ImmunoResearch Labs) at RT for 3 hours, followed by incubation with anti-rabbit Plus and anti-goat Minus probes at 37°C for 1 hour, ligation at 37°C for 30 min, and amplification at 37°C for 100 min. After washing, the tissue sections were incubated with the detection reagent at RT for 1 hr, and the signals were developed with substrate at RT for 5 to 15 min and counterstained with nuclear staining solution. The slides were mounted for imaging.

### *In Vitro* Fibrillation Assay

Reactions were carried out in non-binding black 96-well microplates (cat#655900, Greiner Bio-One North America Inc.). Each well contains 10 nM recombinant proteins or 100 nM c-MYC peptides with 10 μM Thioflavin T (ThT) in 1xPBS. Plates were incubated at 37°C with gentle shaking at 220 rpm in an Eppendorf ThermoMixer C (Eppendorf North America). ThT fluorescence was measured at Ex 450 nm/Em 500 nm using a CLARIOstar microplate reader (BMG LABTECH).

### A11^+^–AO ELISA

Following *in vitro* fibrillation assays, reaction solutions in multiple wells of 96-well microplates were collected and centrifuged at 10,000 rpm for 5 min. The collected supernatants were coated on 96-well UltraCruz^®^ ELISA microplates (cat#sc-204463, Santa Cruz Biotechnology Inc.), 100 μl per well, at 4°C overnight. Plates were washed with 1xPBS and blocked with 5% nonfat milk in 1xTBS-T at RT for 1 hour. Following blocking, diluted A11 antibody (1:1000) in the blocking buffer was added 100 μl/well and incubated at 4°C overnight with gentle shaking. After washing with 1xTBS-T, each well was incubated with 100 μl of goat anti-rabbit IgG) poly-HRP conjugates (cat#PI32260, ThermoFisher Scientific, 1:1000 dilution in the blocking buffer) at RT for 1 hour. Following washing, 100 μl of 1-Step Ultra TMB-ELISA substrates was added to each well. The plates were incubated at RT on a shaker for 2–5 min, followed by measuring the OD at 650 nm using a SpectraMax iD5 multi-mode microplate reader (Molecular Devices).

### Transmission Electron Microscopy

Recombinant proteins were first desalted and reconstituted in 1xPBS. Protein concentrations were adjusted to 0.5 μM with 1xPBS in 96-well non-binding microplates and incubated at 37°C with gentle shaking at 400 rpm in an Eppendorf ThermoMixer C (Eppendorf North America). Following incubation for 5 days, 10 μl of each reaction solution was applied on a piece of parafilm. Then, a water-treated 400-mesh carbon-coated copper grid (cat#CF400-Cu-50, Electron Microscopy Sciences Inc.) was immersed in the droplet of sample suspensions for 1 min, and the remaining liquid was wicked away. The grids were immediately incubated with a drop of 2% uranyl acetate solution (cat#22400–2, Electron Microscopy Sciences Inc.) for 1 min. After wicking, the grids were air-dried and examined under a Hitachi HT7800 transmission electron microscope (Hitachi, Ltd. Group, Japan) operating at 120 kV.

### Transfection and c-MYC dual reporter assays

All plasmids were transfected with TurboFect^™^ transfection reagents (cat# R0531, Thermo Fisher Scientific). HeLa cells were co-transfected with pMYC-SEAP and pCMV-Gaussia luciferase (GLuc) reporter plasmids, along with various indicated plasmids for 24 hr. Reporter activities in culture media were measured. SEAP and GLuc activities in culture supernatants were quantitated using a NovaBright Phospha-Light EXP Assay Kit (cat# N10578, Thermo Fisher Scientific) for SEAP and a Pierce^™^ Gaussia Luciferase Glow Assay Kit (cat#16160, Thermo Fisher Scientific), respectively. Luminescence signals were measured by a CLARIOstar microplate reader (BMG LABTECH). SEAP activities were normalized against GLuc activities.

### Detection of Apoptosis

NIH3T3 cells were seeded in 6-well culture plates and transfected with plasmid DNAs for 24 hours. The cell culture medium was refreshed with 0.5% serum-containing DMEM for 20 hours. Cells were then collected by trypsin-EDTA digestion, washed and fixed with 4% formaldehyde at RT for 30 min. Fixed cells were subsequently permeabilized with 0.3% Triton X-100 in 1xPBS at RT for 10 min. Following wash with 1xPBS, cell pellets were resuspended and incubated with 150 μL of rabbit anti-cleaved caspase-3 Abs (cat#9661, Cell Signaling Technology, 1:200 diluted with 5% BSA in 1xPBS) at 4°C overnight. Subsequently, cells were washed and incubated with Alexa Fluor 647-conjugated goat anti-rabbit IgG (H+L) (cat# A-21245, Thermo Fisher Scientific, 1:700 diluted with 5% BSA in 1xPBS) for 1h at RT. Following washing and resuspending in 1xPBS, cells were subjected to quantification by Flow Cytometry. For detection of Alexa Fluor 647, a BD LSRFortessa^™^ flow cytometer (BD Biosciences, San Jose, CA) with red laser excitation at 640 nm and emission detector bandpass filter of 670/30 nm was used. Data were collected using BD FACS Diva software version 8.0.1.

## QUANTIFICATION AND STATISTICAL ANALYSIS

Statistical analyses were performed using Prism 10 (GraphPad Software). All results are expressed as mean±SD, mean±SEM, or median±IQR. The statistical significance is defined as: *p<0.05, **p<0.01; ***p<0.001; ****p<0.0001; n.s.: not significant. The *in vitro* and *ex vivo* experiments were not randomized.

## Supplementary Material

Supplement 1
