# c-MYC is an aggregation-prone, amyloidogenic protein

**DOI:** 10.64898/2026.03.12.711438

**Published:** 2026-04-09

**Authors:** Ling Lin, Kun-Han Chuang, Chengkai Dai

**Affiliations:** 1 Mouse Cancer Genetics Program, Center for Cancer Research, National Cancer Institute, Frederick, MD 21702, USA; 2 Current address: School of Basic Medical Sciences, Fujian Medical University, Fuzhou, Fujian 350108, China; 6 Lead contact

## Abstract

The transcription factor c-MYC is a central regulator of diverse biological processes and an oncogenic driver. Paradoxically, c-MYC also exhibits intrinsic tumor-suppressive activity through apoptosis, a phenomenon known as the “c-MYC paradox”. Here, we identify c-MYC as an intrinsically amyloidogenic protein. Under proteotoxic stress, c-MYC becomes detergent-insoluble aggregates. Notably, even in the absence of stress, c-MYC assembles into soluble amyloid-like oligomers in human cancer tissues and Alzheimer’s disease brains. *In vitro*, recombinant c-MYC proteins spontaneously form amyloid oligomers and protofibrils. In contrast, its obligate dimerization partner MAX is non-amyloidogenic and suppresses c-MYC amyloidogenesis. Mapping studies identify two short linear sequences within intrinsically disordered regions that confer amyloidogenicity. Importantly, the amyloid state of c-MYC induces apoptosis largely independently of transcription. Thus, these findings reveal a previously unrecognized amyloidogenic property of c-MYC, which contributes to its tumor-suppressing activity. Conceptually, c-MYC amyloidogenesis may serve as a built-in failsafe mechanism to restrain its oncogenic potential.

## INTRODUCTION

The bHLH/ZIP transcription factor c-MYC plays vital roles in human biology, functioning as both an oncogenic driver and a pluripotency-reprogramming factor.^1,2^ Through interaction with MYC-associated factor X (MAX), MYC/MAX dimers bind to the canonical E-box (5’-CACGTG-3’) element or its variants and regulate the transcription of approximately 10–15% of the human genome.^1,3^ Dimerization with MAX is widely regarded as a prerequisite for c-MYC transcriptional activity.^4^ In primary, non-transformed cells, c-MYC protein undergoes rapid turnover and is maintained at low levels; in contrast, its expression is frequently elevated in human cancers due to gene amplification, chromosomal translocation, or increased protein stability.^5^ Despite being a prominent oncoprotein, c-MYC can paradoxically induce apoptosis, an intrinsic tumor-suppressive mechanism, particularly under conditions of cellular stress or high expression.^6,7^ This pro-apoptotic activity has largely been attributed to its transcriptional function.^8,9^

Proteins, including c-MYC, are normally functional in soluble and properly folded conformations; however, proteotoxic stress and genetic mutations can promote protein misfolding and aggregation, leading to loss of function and cytotoxicity. Amyloids, a distinct class of protein aggregates enriched in cross-b-sheet structures, are strongly associated with human diseases, particularly neurodegenerative disorders.^10^ Two fluorescent dyes, Congo red (CR) and thioflavin T (ThT), are widely used to detect amyloids through direct binding.^11^ In addition, the conformation-specific antibody A11 has become a valuable tool for recognizing soluble prefibrillar amyloid oligomers (AOs), a highly neurotoxic conformer independent of primary amino acid sequences.^12^ Beyond its diagnostic utility, CR has also been shown to inhibit amyloid formation.^13,14^

Here, we discover that c-MYC, but not its obligate dimerization partner MAX, forms detergent-insoluble aggregates upon heat shock (HS). Furthermore, in human cancer tissues and, unexpectedly, in Alzheimer’s disease (AD) brains, c-MYC assembles into detergent-soluble oligomers detectable by the A11 antibody even in the absence of HS. Notably, this aggregation and oligomerization can be inhibited by CR. *In vitro*, recombinant c-MYC, but not MAX, proteins spontaneously form AOs and protofibrils, indicating its intrinsic amyloidogenic property. MAX, intriguingly, suppresses c-MYC amyloidogenesis. Screening a synthetic peptide library derived from human c-MYC identified two intrinsically disordered short linear fragments (aa20–39 and aa210–228) as amyloidogenic. Alanine-scanning mutagenesis further revealed that a large proportion of residues within these two fragments contribute to their amyloid-forming capacity. Unexpectedly, a transcription-deficient c-MYC mutant lacking the C-terminal MAX-dimerization and DNA-binding domain retains the ability to induce apoptosis. Importantly, deletion of these two amyloidogenic regions markedly attenuates this apoptotic activity. Taken together, these findings identify c-MYC as an aggregation-prone, intrinsically amyloidogenic protein. Functionally, the amyloid state of c-MYC promotes apoptosis independently of transcriptional activity, revealing a previously unrecognized mechanism underlying its tumor-suppressive function. These results provide new insights into the longstanding c-MYC paradox and reveal its complex implications in human pathologies, including cancer and neurodegeneration.

## RESULTS

### c-MYC acquires amyloid-like properties under stress and pathological conditions

We recently reported that, under non-stressed conditions, heat shock factor 1 (HSF1) potentiates the c-MYC-mediated transcription through physical interactions, representing a non-canonical transcriptional action of HSF1.^15^ We therefore investigated how this c-MYC-HSF1 interaction is affected under proteotoxic stress conditions, such as HS, during which HSF1 is required to initiate the canonical heat-shock, or proteotoxic stress, response (HSR/PSR).^16,17^

**Figure 1:**
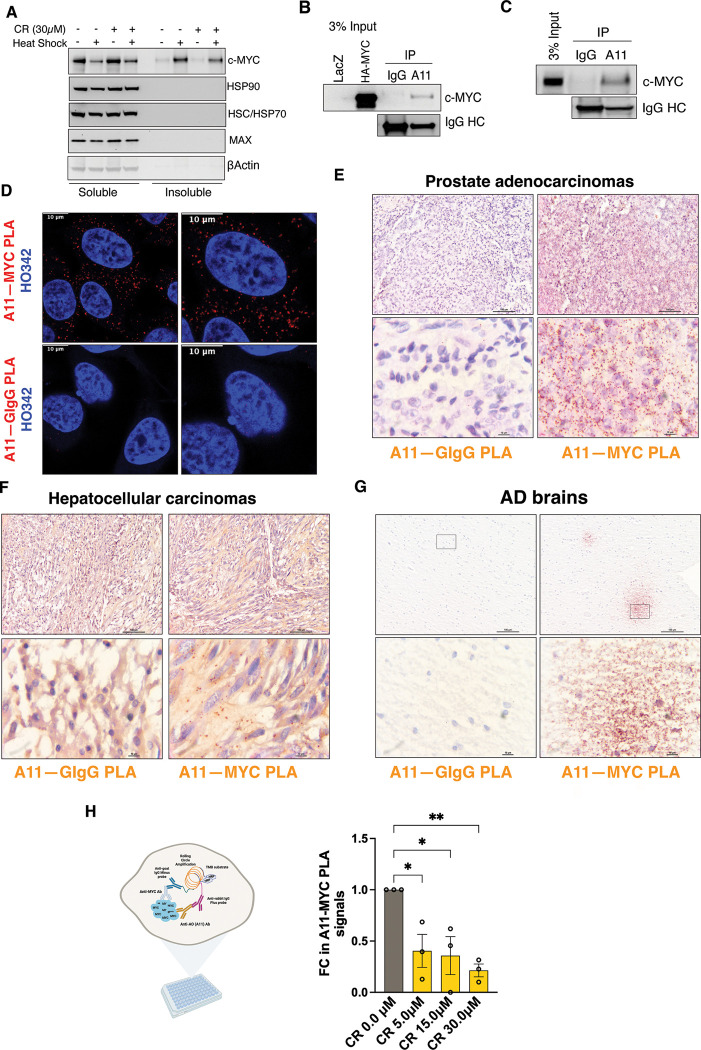
c-MYC becomes detergent-insoluble and displays amyloid-like properties under proteotoxic stress and pathological conditions. (A) Detection of detergent-soluble and - insoluble c-MYC proteins by immunoblotting in HeLa cells pre-treated with 30 μM CR, followed by HS at 43°C for 30 min (representative images of three independent experiments). (B) and (C) Immunoprecipitation of either exogenously expressed HA-c-MYC or endogenous c-MYC proteins in HeLa cells by A11 antibodies (representative images of three independent experiments). (D) Detection of endogenous c-MYC proteins in HeLa cells by PLA using both the goat anti-c-MYC Ab and the rabbit anti-AO (A11) Ab. Nuclei are counterstained with Hoechst 33342. Scale bar: 10 μm. (E)-(G) Detection of endogenous c-MYC proteins in human prostate adenocarcinoma, hepatocellular carcinoma, and Alzheimer’s disease brain tissues by brightfield PLA using both the goat anti-c-MYC Ab and the rabbit anti-AO (A11) Ab. Scale bar: 100 μm for low magnification and 10 μm for high magnification. (H): Quantitation of endogenous c-MYC recognized by both the goat anti-c-MYC Ab and the rabbit anti-AO (A11) Ab through In-Cell PLA ELISA in HeLa cells treated with CR (mean ± SD, n=3 independent experiments, One-way ANOVA).

In HeLa cells, transient HS unexpectedly resulted in a marked reduction in detergent-soluble c-MYC, accompanied by a corresponding increase in detergent-insoluble c-MYC (Figure 1A), indicating stress-induced protein aggregation. In contrast, its obligate dimerization partner MAX and major cellular chaperones, including HSP90 and HSC/HSP70, remained soluble. Of note, c-MYC aggregation was partially blocked by CR (Figure 1A). Although CR is widely used as an amyloid-binding dye and inhibitor of amyloidogenesis, it may also exert broader effects on protein aggregation.

To determine whether c-MYC forms amyloid-like aggregates, we utilized the conformation-specific A11 antibody, which has been widely applied to recognize soluble prefibrillar AOs.^12^ In HeLa cells, A11 immunoprecipitation captured both exogenous HA-c-MYC and endogenous c-MYC proteins in the absence of HS (Figures 1B and 1C). These findings were further confirmed by *in situ* Proximity Ligation Assay (PLA), ^18^ which detects protein-protein interactions or two distinct epitopes within the same protein (Figure 1D). The PLA signals were quantified by flow cytometry (Figure S1A). Notably, these PLA signals were predominantly localized in the cytoplasm (Figure 1D), suggesting that these c-MYC conformers are transcriptionally inactive. The presence of such conformers was not an artifact of cell culture, as A11^+^-c-MYC oligomers were readily detected in both primary and xenografted human cancer tissues (Figures 1E, 1F, and S1B). Intriguingly, similar c-MYC oligomers were also observed in human AD brain tissues (Figure 1G), where they resembled diffuse Aβ plaques. Importantly, the genesis of A11^+^-c-MYC oligomers was inhibited by CR treatment (Figures 1H and S1C), further corroborating their amyloidogenic nature. Collectively, these results demonstrate that c-MYC proteins, at least under proteotoxic stress and in pathological contexts, can acquire amyloid-like properties.

### c-MYC is intrinsically amyloidogenic

It should be noted that both immunoprecipitation and PLA staining cannot distinguish whether c-MYC interacts with pre-existing AOs or itself adopts an amyloid conformation. To address this, we performed *in vitro* assays using purified recombinant proteins.

**Figure 2:**
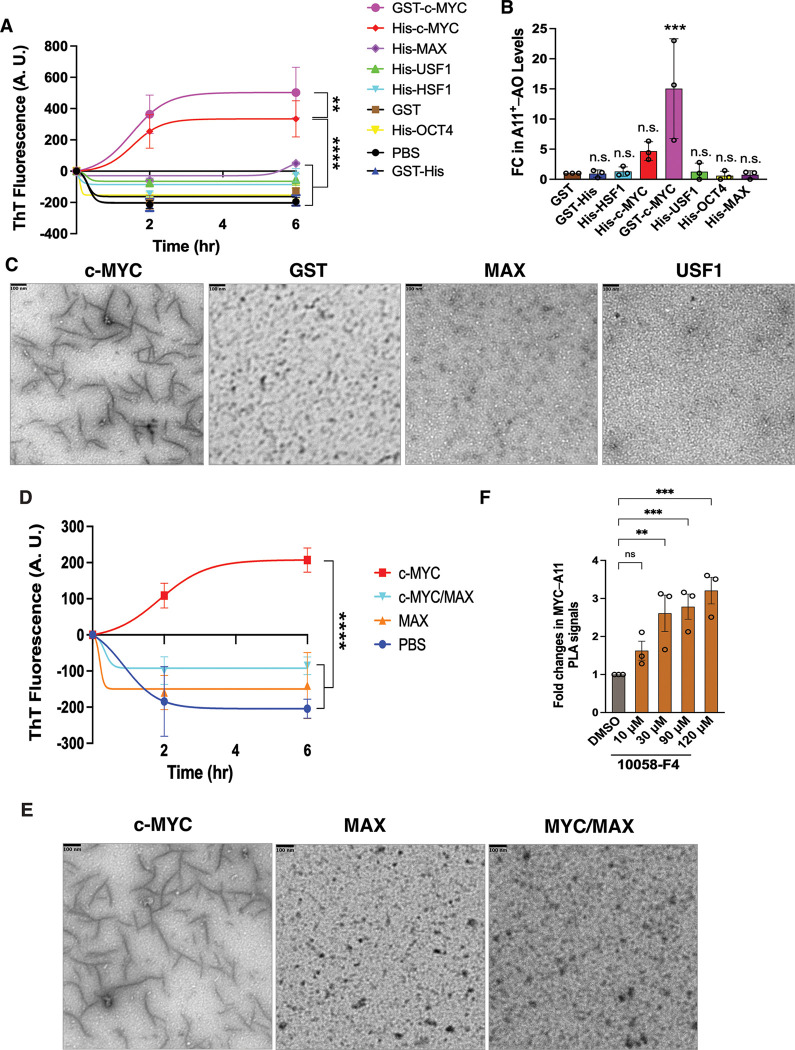
c-MYC is intrinsically amyloidogenic. (A) *In vitro* fibrillation assays using recombinant c-MYC proteins (mean ± SEM, n=3 independent experiments, Two-way ANOVA). (B) ELISA quantitation of A11^+^-AOs generated by recombinant c-MYC proteins *in vitro* (mean ± SD, n=3 independent experiments, One-way ANOVA). (C) TEM visualization of protofibrils formed by recombinant c-MYC proteins *in vitro* (representative images of three independent experiments). Scale bars: 100 nm. (D) *In vitro* fibrillation assays using recombinant c-MYC /MAX dimers (mean ± SEM, n=3 independent experiments, Two-way ANOVA). (E) TEM visualization of recombinant c-MYC/MAX dimers *in vitro* (representative images of three independent experiments). Scale bars: 100 nm. (F) Quantitation of endogenous c-MYC AOs in HeLa cells treated with 10058-F4 by In-Cell PLA ELISA (mean ± SD, n=3 independent experiments, One-way ANOVA).

In classic *in vitro* fibrillation assays, recombinant c-MYC proteins obtained by two independent sources spontaneously assembled into amyloid fibrils, as detected by ThT binding (Figure 2A). This sharply contrasted with GST and a panel of recombinant transcription factors, including HSF1, USF1, OCT4, and MAX (Figure 2A). Of note, neither MAX, the obligate dimerization partner of c-MYC, nor USF1, another member of the c-MYC family, formed amyloid fibrils, highlighting the specific amyloidogenicity of c-MYC. In addition to fibrils, recombinant c-MYC proteins also formed soluble AOs, as recognized by the A11 antibody (Figure 2B). Likely due to the solubilizing effect of the GST tag, GST-c-MYC proteins generated substantially higher levels of AOs. Transmission electron microscopy (TEM) further confirmed the assembly of protofibrils by recombinant c-MYC proteins (Figure 2C). Collectively, these *in vitro* data demonstrate that c-MYC is intrinsically amyloidogenic.

Considering that c-MYC physiologically dimerizes with non-amyloidogenic protein MAX,^4^ we next examined how this interaction influences c-MYC amyloidogenesis. *In vitro* fibrillation assays showed that recombinant c-MYC/MAX heterodimers failed to form amyloid fibrils, in stark contrast to c-MYC proteins alone (Figures 2D and 2E). Consistently, disruption of MYC/MAX dimerization in HeLa cells using the small molecule 10058-F4 resulted in increased levels of A11^+^–c-MYC oligomers, despite a reduction in detergent-soluble c-MYC proteins (Figures 2F, S2A, and S2B).^19^ Together, these results indicate that c-MYC is intrinsically amyloidogenic and that dimerization with MAX suppresses its amyloid formation.

### Deciphering the intrinsic amyloidogenic regions of c-MYC

**Figure 3:**
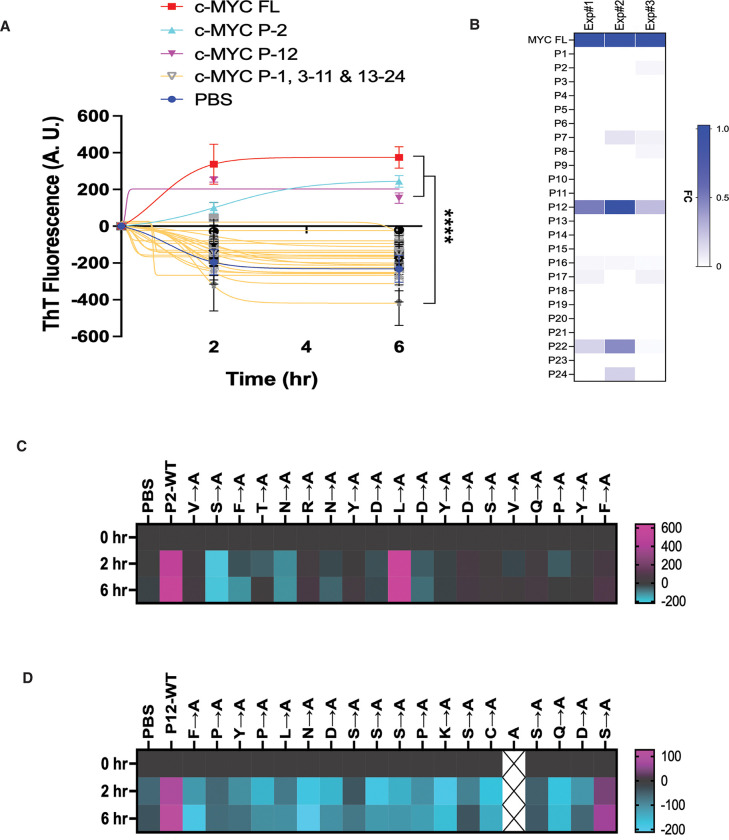
Decoding the amyloidogenicity of c-MYC *in vitro*. (A) *In vitro* fibrillation assays using the human c-MYC peptide library (mean ± SEM, n=3 independent experiments, Two-way ANOVA). (B) ELISA quantitation of A11^+^-AOs generated by synthetic c-MYC peptides. Results of three independent experiments are presented as a heatmap. FC: fold change. (C) and (D) *In vitro* fibrillation assays using the P2 and P12 peptides that are subjected to alanine scan. Results are presented as heatmaps using the averages of three independent experiments.

To systematically map the amyloidogenic regions of c-MYC, we designed a synthetic peptide library spanning the human c-MYC protein, with each peptide comprising 19 non-overlapping amino acids. This technique has previously been applied to delineate protein-protein interaction interfaces.^15^ Among the 24 peptides tested, only two—P2 (aa20–38) and P12 (aa210–228)—exhibited evident amyloidogenic activity, like the full-length c-MYC protein, as determined by *in vitro* fibrillation assays (Figure 3A). Of interest, only P12 consistently formed A11^+^-AOs (Figure 3B). Both P2 and P12 are located within intrinsically disordered regions (IDRs) of c-MYC (Figure S3A).

To further pinpoint residues critical for amyloid formation, both P2 and P12 were subjected to alanine-scanning mutagenesis. *In vitro* fibrillation assays revealed that substitution of individual residues with alanine generally led to a marked reduction in amyloid fibril formation (Figures 3C and 3D). These results indicate that short linear sequences within intrinsically disordered regions constitute the amyloidogenic determinants of c-MYC. Moreover, multiple residues within these regions collectively contribute to its amyloidogenicity.

### Biological implications of c-MYC amyloidogenesis

**Figure 4:**
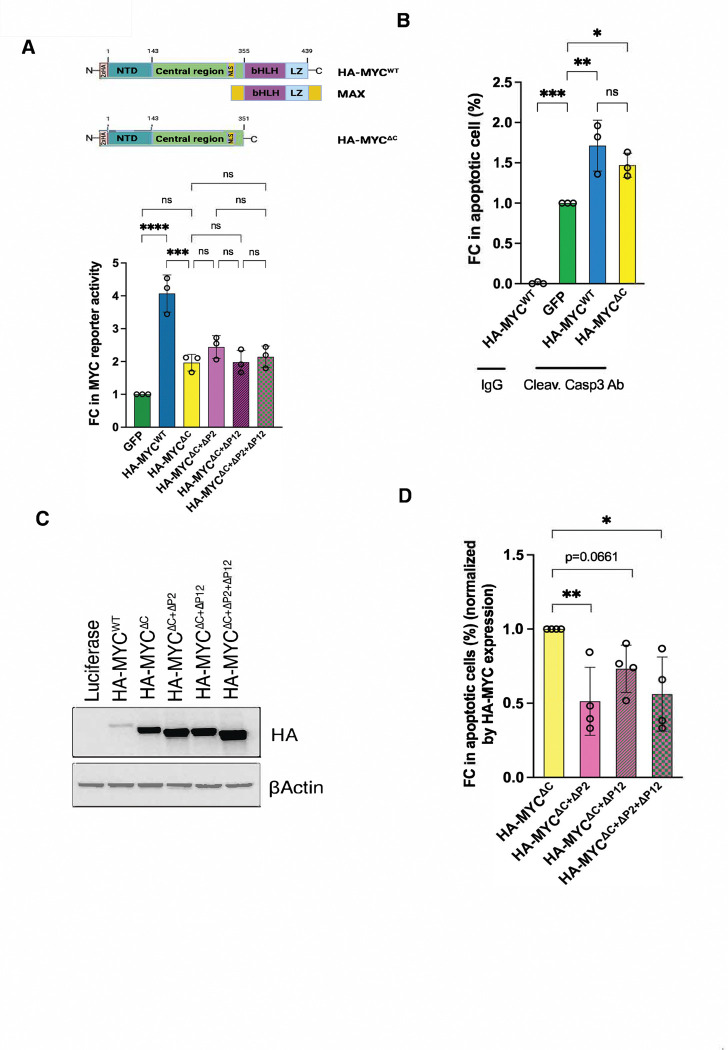
The amyloidogenesis of c-MYC contributes to its intrinsic tumor suppressor activity. (A) Measurements of the transcriptional activities of HA-MYC^WT^ and HA-MYC^ΔC^ in HeLa cells using the dual MYC reporter system (mean ± SD, n=3 independent experiments, One-way ANOVA). (B) Quantitation of apoptosis induced by transient expression of HA-MYC^WT^ or HA-MYC^ΔC^ in serum-starved NIH3T3 cells by flow cytometry using anti-cleaved caspase 3 Abs (mean ± SD, n=3 independent experiments, One-way ANOVA). (C) Detection of HA-MYC expression in serum-starved NIH3T3 cells by immunoblotting (representative images of four independent experiments). (D) Quantitation of apoptosis induced by transient expression of HA-MYC^ΔC^ and its deletion mutants in serum-starved NIH3T3 cells by flow cytometry using anti-cleaved caspase 3 Abs. The results are normalized by their expression levels (mean ± SD, n=4 independent experiments, One-way ANOVA).

Protein aggregates, particularly amyloids, are often cytotoxic. We therefore hypothesized that c-MYC amyloidogenesis may contribute to apoptosis, thereby underlying its tumor-suppressive activity. It is well established that c-MYC can induce apoptosis under stress conditions, including serum starvation and DNA damage, or upon elevated expression. ^6–9^ To distinguish transcription-dependent from amyloid-dependent apoptosis, we generate a transcription-deficient HA-c-MYC^ΔC^ mutant lacking the C-terminal DNA-binding and MAX-dimerization domains, while retaining the amyloidogenic regions (Figure 4A). As expected, overexpression of HA-c-MYC^WT^ induced apoptosis in serum-starved NIH-3T3 cells, as indicated by caspase 3 cleavage (Figure 4B). Surprisingly, so did HA-c-MYC^ΔC^ mutants (Figures 4B), suggesting the existence of a transcription-independent mechanism. To determine whether amyloidogenesis contributes to this apoptotic activity, the two identified amyloidogenic regions were deleted from HA-c-MYC^ΔC^. Deletions of these regions affected protein expression levels (Figure 4C); therefore, apoptotic effects were normalized accordingly. Deletion of the P2 region markedly diminished apoptosis, whereas deletion of the P12 region had a more modest effect (Figure 4D). Importantly, removal of these amyloidogenic regions did not further impair the residual transcriptional activity of HA-c-MYC^ΔC^ mutants (Figure 4A), yet significantly diminished its ability to induce apoptosis (Figure 4D), thereby decoupling apoptosis induction from transcriptional activity. In aggregate, these findings indicate that c-MYC amyloidogenesis promotes cytotoxicity and may contribute to its intrinsic tumor suppressor function.

## DISCUSSION

### c-MYC is an aggregation-prone and amyloidogenic protein

Our combined *ex vivo* and *in vitro* analyses provide strong evidence that c-MYC is an aggregation-prone, intrinsically amyloidogenic protein. This conclusion is supported by multiple independent observations, including inhibition of c-MYC aggregation and amyloidogenesis by CR, ThT binding indicative of c-MYC amyloid fibril formation, recognition of c-MYC AOs by the conformation-specific A11 antibody, and direct visualization of c-MYC protofibrils by TEM. Importantly, we identified two intrinsically disordered short linear sequences as the primary determinants of c-MYC amyloidogenicity. Although both regions assembled into amyloid fibrils *in vitro*, as evidenced by ThT binding, only one region (P12) consistently generated A11^+^–AOs. It is possible that P2 region forms a distinct class of AOs that cannot be recognized by the A11 antibody.

### MAX suppresses c-MYC amyloidogenesis

A notable finding of this study is that MAX, the obligate dimerization partner of c-MYC and itself non-amyloidogenic, suppresses c-MYC amyloid formation. This observation suggests that in primary non-transformed cells—where c-MYC expression is low and MAX is sufficiently available for dimerization—c-MYC amyloidogenesis is effectively restrained.

In contrast, a markedly different scenario is likely to occur in cancer cells, where *c-MYC* is frequently overexpressed and its expression levels are poorly correlated with those of *MAX* (Figures S4A, S4B, and S4C). Conceivably, while c-MYC is positively selected for its oncogenic activity, MAX is not subject to the same selective pressure, in part due to its tumor-suppressing functions through dimerization with MXD family proteins. ^20,21^ This imbalance in c-MYC and MAX expression may permit or even promote c-MYC amyloidogenesis in human cancers.

### New insights into the c-MYC paradox

The “c-MYC paradox” depicts an intriguing dichotomy, where c-MYC acts as an oncogenic driver that also possesses intrinsic tumor suppressor activity. This pro-apoptotic function, traditionally, has been attributed exclusively to the transcriptional activity of c-MYC. Our findings now demonstrate that the amyloid state of c-MYC can induce apoptosis in a largely transcription-independent manner. Thus, c-MYC can exert tumor-suppressive effects through both transcription-dependent and transcription-independent mechanisms, likely influenced by its cellular abundance.

Given the fundamental roles of c-MYC in cellular physiology, its expression is tightly regulated under normal conditions; disruption of this regulation is strongly associated with pathogeneses, particularly malignancy. Conceptually, the amyloidogenic conversion of c-MYC may serve as a built-in failsafe mechanism that limits its oncogenic potential by promoting cytotoxicity.

### Implications of c-MYC amyloidogenesis in cancer and neurodegeneration

We previously described the phenomenon of “tumor-suppressive amyloidogenesis”, ^14^ arising from widespread proteomic instability in cancer. The present findings further identify c-MYC as a prototypical example of tumor-associated amyloids. Given the cytotoxic nature of amyloid conformers, they must be contained to avert tumor-suppressing effects in cancer cells. Our prior work demonstrated that HSF1, the master regulator of the HSR/PSR, can physically neutralize toxic AOs. ^22^ Notably, HSF1 happens to interact with c-MYC. ^15^ It is therefore plausible that, in cancer cells, HSF1 may help counteract these c-MYC amyloids. Consistent with this notion, *HSF1* and *c-MYC* expression levels are significantly positively correlated in human cancers.^15^

Beyond cancer, our data indicate that c-MYC amyloidogenesis also occurs in human AD brain tissues. In fact, previous studies have reported elevated MYC expression in human AD brains.^23,24^ Moreover, neuronal overexpression of *c-MYC* induced neurodegeneration in mice, an effect attributed to neuronal cell cycle re-entry. ^25^ Our findings, nevertheless, raise the possibility that c-MYC amyloidogenesis may contribute to neurodegenerative processes, a hypothesis that warrants further investigation.

### EXPERIMENTAL METHODS

#### Cell Lines and Reagents

HeLa (female) cells and NIH3T3 (male) cells were purchased from ATCC. They were authenticated by ATCC by STR profiling. All cell cultures were maintained in DMEM supplemented with 10% HyClone bovine growth serum and 1% penicillin–streptomycin. Cells were maintained in an incubator with 5% CO2 at 37 °C. All cell lines were routinely tested for mycoplasma contamination using MycoAlert Mycoplasm Detection kits.

Recombinant proteins were all purchased commercially, including c-MYC/MAX complexes (cat#81087, Active Motif Inc.), His-HSF1 (cat#ADI-SPP-900-F, Enzo Life Sciences Inc.), GST-c-MYC (cat#M86–30G, Sino Biological US Inc.), His-c-MYC (cat#230–00580-100, RayBiotech Inc.), GST (cat#G52–30U-50, SignalChem Inc.), His-GST (cat#RPT0002, ABclonal Inc.), His-MAX (cat#81017, Active Motif Inc.), His-USF1 (cat#228–21891-2, RayBiotech Inc.), and His-OCT4 (cat#228–21139-2, RayBiotech Inc.).

All antibodies were purchased commercially, including rabbit polyclonal anti-amyloid oligomer (A11) Ab (cat#SPC-506, StressMarq Biosciences Inc.), rabbit monoclonal anti-c-MYC/N-MYC (D3N8F) Ab (cat#13987, Cell Signaling Technology), rabbit monoclonal ani-MAX (E6F6Y) Ab (cat#17471, Cell Signaling Technology), mouse monoclonal anti-HSC/HSP70 (W27) Ab (cat#sc-24, Santa Cruz Biotechnology Inc.), mouse monoclonal anti-HSP90a/b (F-8) Ab (cat#sc-13119, Santa Cruz Biotechnology Inc.), rabbit monoclonal anti-cleaved caspase 3 (Asp175) (D3E9) Ab (cat#9579, Cell Signaling Technology), goat polyclonal anti-c-MYC Ab (cat#AF3696, R&D Systems Inc.), mouse monoclonal anti-HA Ab (cat#901513, BioLegend Inc.), mouse monoclonal anti-bActin (GT5512) Ab (cat#GTX629630, GeneTex Inc.), normal rabbit IgG (cat#02–6102,ThermoFisher Scientific Inc.), Peroxidase AffiniPure Goat Anti-Rabbit IgG (H+L) (cat#111–035-144, Jackson ImmunoResearch Inc.), Peroxidase AffiniPure Goat Anti-Mouse IgG (H+L) (cat#115–035-003, Jackson ImmunoResearch Inc.), and Peroxidase IgG Fraction Monoclonal Mouse Anti-Goat IgG, light chain specific (cat#205–032-176, Jackson ImmunoResearch Inc.).

All chemicals were purchased commercially, including Thioflavin T (ThT) (cat# AC211760050, ThermoFisher Scientific Inc.), Congo red (CR) (cat#C580–25, ThermoFisher Scientific Inc.), Janus Green B (cat# AC191680050, Fisher Scientific LLC), and 10058-F4 (cat#T3048, TargetMol Chemicals Inc.).

pcDNA-GFP and pcDNA3–2xHA-MYC were gifts from Martine Roussel (Addgene plasmid#74165 and 74164, respectively). pcDNA3–2xHA-MYC^ΔC^, 2xHA-MYC^ΔC+ΔP2^, 2xHA-MYC^ΔC+ΔP12^, and 2xHA-MYC^ΔC+ΔP2+ΔP12^ were generated using the Q5 Site-Directed Mutagenesis kit (cat#E0554S, New England Biolabs Inc.). pMYC-SEAP was purchased from Clontech Laboratories (Cell signaling pathway profiling systems, cat#631910) and pCMV-Gaussia Luc was purchased from Thermo Fisher Scientific (cat#16147).

The c-MYC peptide library and alanine scan libraries were custom synthesized by GenScript USA Inc.

Paraffin sections of human liver cancer (cat#HuCAT081) and prostate cancer (cat#HuCAT371) were purchased from TissueArray.com LLC. Paraffin sections of human Alzheimer’s disease brains (cat#CSA0225P) were purchased from American MasterTech Scientific. Paraffin sections of human A2058 melanomas were prepared from xenografted tumors described previously. ^14^

#### Soluble and Insoluble Fractionation

HeLa cells were treated with Congo red for 24 hours, followed by incubation at 43°C for 30 min. Cells were then trypsinized, washed, and resuspended in 1xPBS for cell counting. Equal numbers of cells were used for soluble and insoluble fractionation. The detailed procedures were described previously. ^22^

#### In-Cell PLA ELISA

To avoid the interference of CR or 10058-F4 in PLA fluorescence detection, In-Cell PLA ELISA was adopted using the Duolink^®^ In Situ Detection Reagent Brightfield (cat#DUO92012, Sigma-Aldrich). The detailed procedures were described previously.^15^ HeLa cells were seeded in 96-well culture plates (7000 cells/well) and treated with CR for 24 hr or 10058-F4 for 30 hr. The goat anti-c-MYC antibody and the rabbit anti-AO (A11) antibody were combined for the PLA, followed by incubation with Duolink^®^ PLA anti-rabbit Plus (cat#DUO92002, Sigma-Aldrich) and anti-goat Minus probes (cat#DUO92006, Sigma-Aldrich). Of note, 1-Step Ultra TMB-ELISA Substrate Solution (cat#34029, ThermoFisher Scientific), instead of the precipitating substrate provided by the kit, was used for the substrate development.

#### Immunoblotting and Immunoprecipitation

Whole-cell protein extracts were prepared in cold cell-lysis buffer (50mM Tris-Cl, pH 7.5, 120mM NaCl, 0.5% NP-40 and 1mM PMSF). Proteins were transferred to nitrocellulose membranes. Following incubation with the blocking buffer (5% non-fat milk in 1x TBS-T) for 1 hour at RT, membranes were incubated with primary antibodies (1:1,000 dilution in the blocking buffer) overnight at 4 °C. After washing with 1xTBS-T for 3 times, membranes were incubated with peroxidase-conjugated secondary antibodies (1: 5000 diluted in the blocking buffer) at RT for 1 hr. Signals were detected using SuperSignal West chemiluminescent substrates (cat#34580 or 34095, Thermo Fisher Scientific Inc.).

For the A11 IP, HeLa cells were seeded for 24 hours or transfected with plasmid DNAs for 24 hours. Following trypsinization and washing in ice-cold 1xPBS, cells were lysed in cell lysis buffer on ice for 30 min and the extracts were centrifuged at 16,813×g for 5 min at 4°C to remove insoluble fractions. Soluble cell lysates (5 mg) were incubated with either rabbit IgG or anti-amyloid oligomers (A11) antibody (5 μg) at 4°C with gentle rotation for overnight. The immune complexes were then recovered with Dynabeads^™^ Protein G (cat#10004D, Life Technologies Corp.) or ChIP-Grade Protein G agarose beads (cat#9007S, Cell Signaling Technology). After washing with the lysis buffer, precipitates were subjected to SDS-PAGE and immunoblotting with either goat anti-c-MYC Abs or mouse anti-HA Abs, followed by incubation with HRP-conjugated bovine anti-goat IgG (cat#805–035-180, Jackson ImmunoResearch Laboratories Inc.) or EasyBlot^®^ anti-mouse IgG (HRP) (cat#GTX221667–01, GeneTex Inc.). Signals were detected using SuperSignal^™^ West Pico PLUS chemiluminescent substrates and documented with an iBright FL1000 Imaging System (ThermoFisher Scientific).

#### *In Situ* Proximity Ligation Assay (PLA)

The general procedures for *in situ* PLA were described previously.^15^ For the specific MYC-A11 *in situ* PLA, the goat anti-c-MYC antibody and the rabbit anti-AO (A11) antibody were used, followed by incubation with Duolink^®^ PLA anti-rabbit Plus and anti-goat Minus probes. Images were captured using a Zeiss LSM780 confocal microscope.

Brightfield *in situ* PLA was performed using the Duolink^®^ In Situ Detection Reagents Brightfield. Paraffin-embedded tissue sections were deparaffinized at 60°C for 1.5 hr and rehydrated. Antigen retrieval was then performed using sodium citrate buffer (pH 6.0) in a vegetable steamer for 25 min. Following permeabilization using 0.3% Triton-X-100 in 1xPBS at RT for 10 min, endogenous peroxidase and alkaline phosphatase activities were quenched using the BLOXALL blocking solution at RT for 10 min and slides were blocked with 1x Duolink blocking solution (cat#DUO82007, Sigma-Aldrich) at 37°C for 1 hr. The tissue slides were incubated with rabbit anti-AO (A11) Abs (1:100) and goat anti-c-MYC Ab (1:100) or goat normal IgG (Jackson ImmunoResearch Labs) at RT for 3 hours, followed by incubation with anti-rabbit Plus and anti-goat Minus probes at 37°C for 1 hour, ligation at 37°C for 30 min, and amplification at 37°C for 100 min. After washing, the tissue sections were incubated with the detection reagent at RT for 1 hr, and the signals were developed with substrate at RT for 5 to 15 min and counterstained with nuclear staining solution. The slides were mounted for imaging.

#### *In Vitro* Fibrillation Assay

Reactions were carried out in non-binding black 96-well microplates (cat#655900, Greiner Bio-One North America Inc.). Each well contains 10 nM recombinant proteins or 100 nM c-MYC peptides with 10 μM Thioflavin T (ThT) in 1xPBS. Plates were incubated at 37°C with gentle shaking at 220 rpm in an Eppendorf ThermoMixer C (Eppendorf North America). ThT fluorescence was measured at Ex 450 nm/Em 500 nm using a CLARIOstar microplate reader (BMG LABTECH). To correct for the differential background signals, the baseline fluorescence readings (*t*_*0*_) were subtracted.

#### A11^+^–AO ELISA

Following *in vitro* fibrillation assays, reaction solutions in multiple wells of 96-well microplates were collected and centrifuged at 10,000 rpm for 5 min. The collected supernatants were coated on 96-well UltraCruz^®^ ELISA microplates (cat#sc-204463, Santa Cruz Biotechnology Inc.), 100 μl per well, at 4°C overnight. Plates were washed with 1xPBS and blocked with 5% nonfat milk in 1xTBS-T at RT for 1 hour. Following blocking, diluted A11 antibody (1:1000) in the blocking buffer was added 100 μl/well and incubated at 4°C overnight with gentle shaking. After washing with 1xTBS-T, each well was incubated with 100 μl of goat anti-rabbit IgG) poly-HRP conjugates (cat#PI32260, ThermoFisher Scientific, 1:1000 dilution in the blocking buffer) at RT for 1 hour. Following washing, 100 μl of 1-Step Ultra TMB-ELISA substrates was added to each well. The plates were incubated at RT on a shaker for 2–5 min, followed by measuring the OD at 650 nm using a SpectraMax iD5 multi-mode microplate reader (Molecular Devices).

#### Transmission Electron Microscopy

Recombinant proteins were first desalted and reconstituted in 1xPBS. Protein concentrations were adjusted to 0.5 μM with 1xPBS in 96-well non-binding microplates and incubated at 37°C with gentle shaking at 400 rpm in an Eppendorf ThermoMixer C (Eppendorf North America). Following incubation for 5 days, 10 μl of each reaction solution was applied on a piece of parafilm. Then, a water-treated 400-mesh carbon-coated copper grid (cat#CF400-Cu-50, Electron Microscopy Sciences Inc.) was immersed in the droplet of sample suspensions for 1 min, and the remaining liquid was wicked away. The grids were immediately incubated with a drop of 2% uranyl acetate solution (cat#22400–2, Electron Microscopy Sciences Inc.) for 1 min. After wicking, the grids were air-dried and examined under a Hitachi HT7800 transmission electron microscope (Hitachi, Ltd. Group, Japan) operating at 120 kV.

#### Transfection and c-MYC dual reporter assays

All plasmids were transfected with TurboFect^™^ transfection reagents (cat# R0531, Thermo Fisher Scientific). HeLa cells were co-transfected with pMYC-SEAP and pCMV-Gaussia luciferase (GLuc) reporter plasmids, along with various indicated plasmids for 24 hr. Reporter activities in culture media were measured. SEAP and GLuc activities in culture supernatants were quantitated using a NovaBright Phospha-Light EXP Assay Kit (cat# N10578, Thermo Fisher Scientific) for SEAP and a Pierce^™^ Gaussia Luciferase Glow Assay Kit (cat#16160, Thermo Fisher Scientific), respectively. Luminescence signals were measured by a CLARIOstar microplate reader (BMG LABTECH). SEAP activities were normalized against GLuc activities.

#### Detection of Apoptosis

NIH3T3 cells were seeded in 6-well culture plates and transfected with plasmid DNAs for 24 hours. The cell culture medium was refreshed with 0.5% serum-containing DMEM for 20 hours. Cells were then collected by trypsin-EDTA digestion, washed and fixed with 4% formaldehyde at RT for 30 min. Fixed cells were subsequently permeabilized with 0.3% Triton X-100 in 1xPBS at RT for 10 min. Following wash with 1xPBS, cell pellets were resuspended and incubated with 150 μL of rabbit anti-cleaved caspase-3 Abs (cat#9661, Cell Signaling Technology, 1:200 diluted with 5% BSA in 1xPBS) at 4°C overnight. Subsequently, cells were washed and incubated with Alexa Fluor 647-conjugated goat anti-rabbit IgG (H+L) (cat# A-21245, Thermo Fisher Scientific, 1:700 diluted with 5% BSA in 1xPBS) for 1h at RT. Following washing and resuspending in 1xPBS, cells were subjected to quantification by Flow Cytometry. For detection of Alexa Fluor 647, a BD LSRFortessa^™^ flow cytometer (BD Biosciences, San Jose, CA) with red laser excitation at 640 nm and emission detector bandpass filter of 670/30 nm was used. Data were collected using BD FACS Diva software version 8.0.1.

### QUANTIFICATION AND STATISTICAL ANALYSIS

Statistical analyses were performed using Prism 10 (GraphPad Software). All results are expressed as mean±SD, mean±SEM, or median±IQR. The statistical significance is defined as: *p<0.05, **p<0.01; ***p<0.001; ****p<0.0001; n.s.: not significant. The *in vitro* and *ex vivo* experiments were not randomized.

## Supplementary Material

Supplement 1
