# Supplementary material for: c-MYC is an aggregation-prone, amyloidogenic protein
Source: bioRxiv. 2026 Apr 9:2026.03.12.711438. Originally published 2026 Mar 13. Preprint. [Version 2] doi: 10.64898/2026.03.12.711438 (PMC13060799; doi:10.64898/2026.03.12.711438)
Supplement: Supplementary file 1 [file media-1.pdf]

LIST OF SUPPLEMENTARY MATERIALS

The supplementary materials include 4 supplementary figures and their legends.

Figure S1: **c-MYC displays amyloid-like properties under pathological conditions, related to Figure 1.**

Figure S2: **c-MYC is intrinsically amyloidogenic, related to Figure 2.**

Figure S3: The amyloidogenic P2 and P12 regions are disordered, related to Figure 3.

Figure S4: The amyloidogenesis of c-MYC contributes to its intrinsic tumor suppressor activity, related to Figure 4 and Discussion.

Figure S1

A

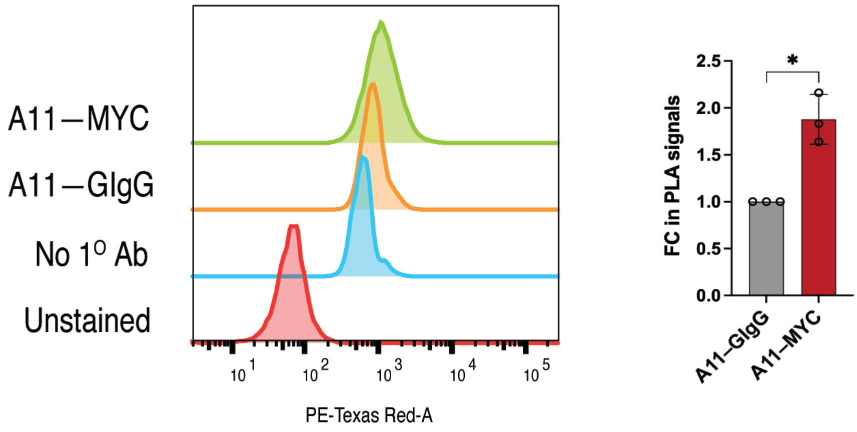

B

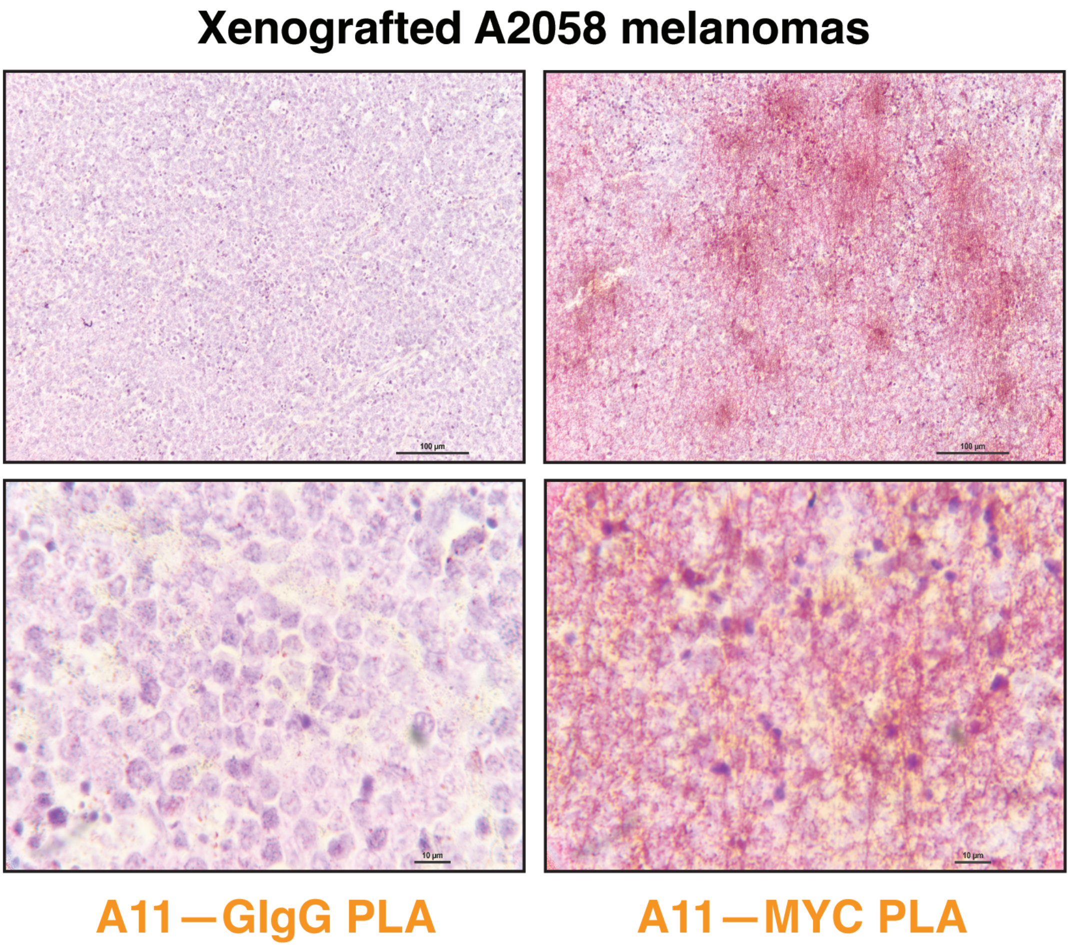

C

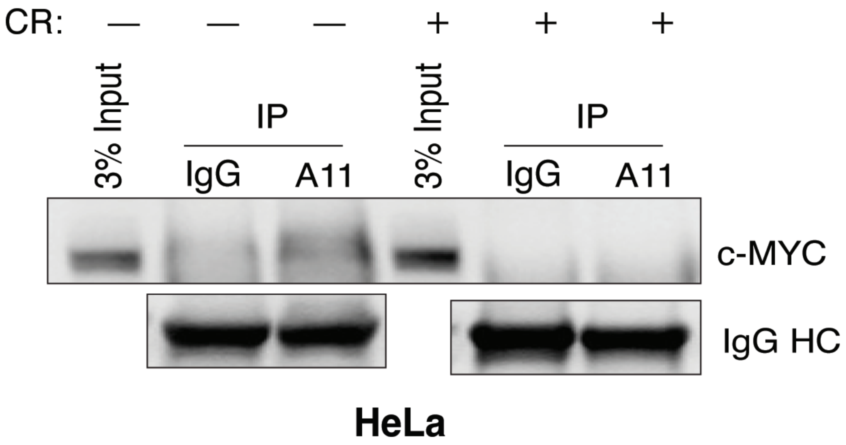

SUPPLEMENTAL FIGURE LEGENDS

Figure S1: c-MYC displays amyloid-like properties under pathological conditions. (A) Quantitation of endogenous c-MYC recognized by both the goat anti-c-MYC Ab and the rabbit anti-AO (A11) Ab through flow PLA (mean ± SD, n=3 independent experiments, Two-tailed Student's *t* test). (B) Detection of endogenous c-MYC proteins in xenografted human A2058 melanomas by brightfield PLA using both the goat anti-c-MYC Ab and the rabbit anti-AO (A11) Ab. Scale bars: 100 μm for low magnification and 10 μm for high magnification. (C) Immunoprecipitation of endogenous c-MYC proteins by A11 antibodies in HeLa cells treated with 30 μM CR.

Figure S2

A

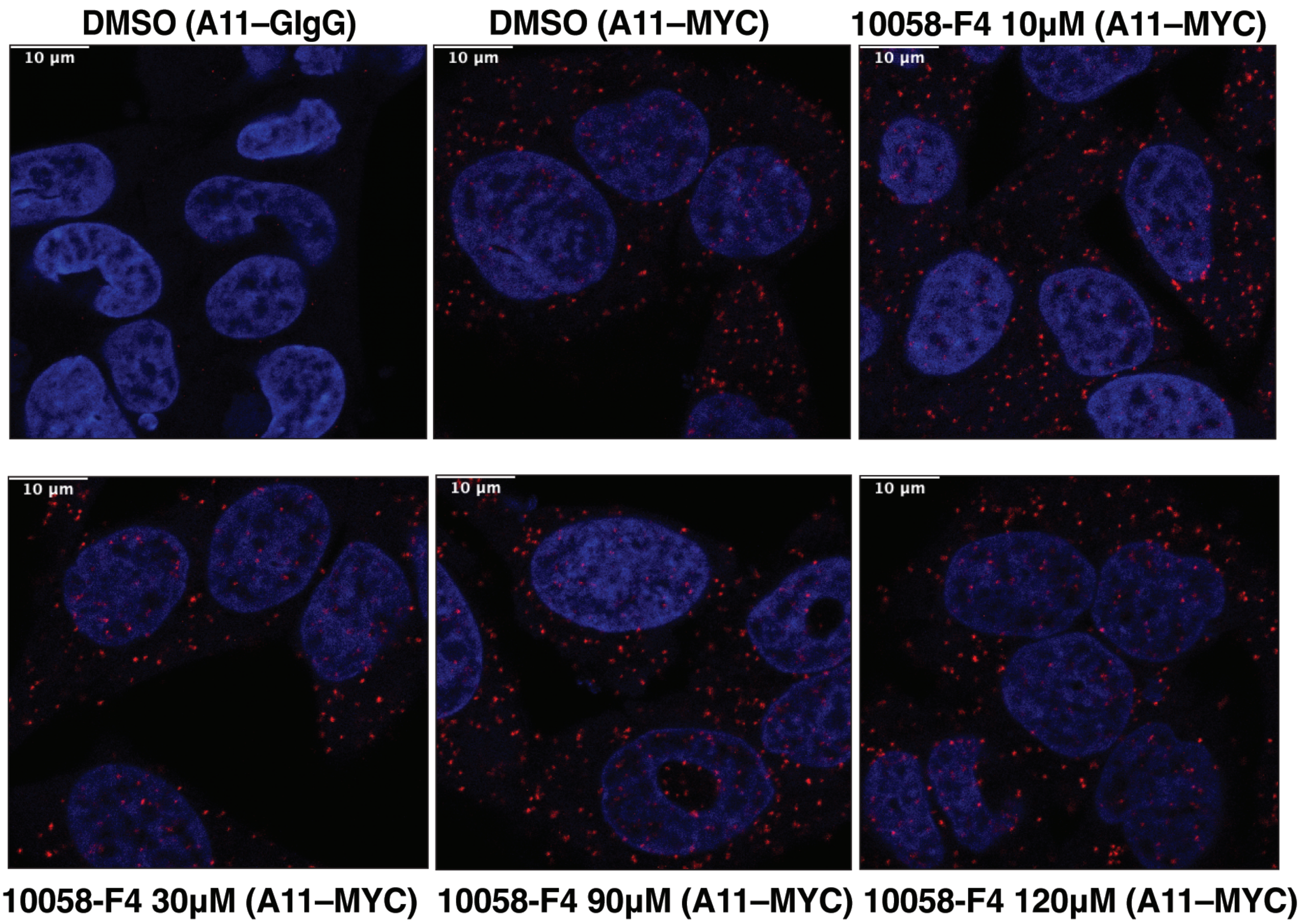
DMSO (A11–GIgG)
DMSO (A11–MYC)
10058-F4 10μM (A11–MYC)
10 μm
10 μm
10 μm
10 μm
10 μm
10 μm
10058-F4 30μM (A11–MYC)
10058-F4 90μM (A11–MYC)
10058-F4 120μM (A11–MYC)

B

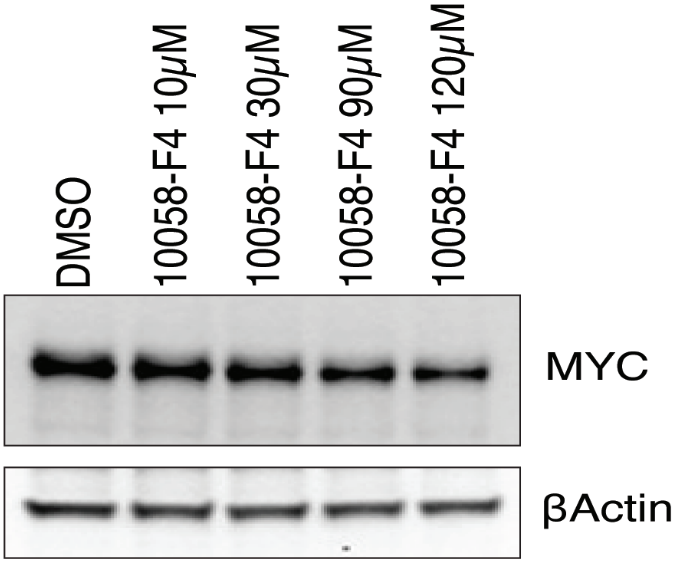
DMSO
10058-F4 10μM
10058-F4 30μM
10058-F4 90μM
10058-F4 120μM
MYC
βActin

Figure S2: c-MYC is intrinsically amyloidogenic. (A) Visualization of endogenous c-MYC AOs in HeLa cells treated with 10058-F4 by fluorescent PLA (representative images of three independent experiments). Scale bars: 10 μm. (B) Detection of c-MYC proteins by immunoblotting in HeLa cells treated with 10058-F4 (representative images of three independent experiments).

Figure S3

A

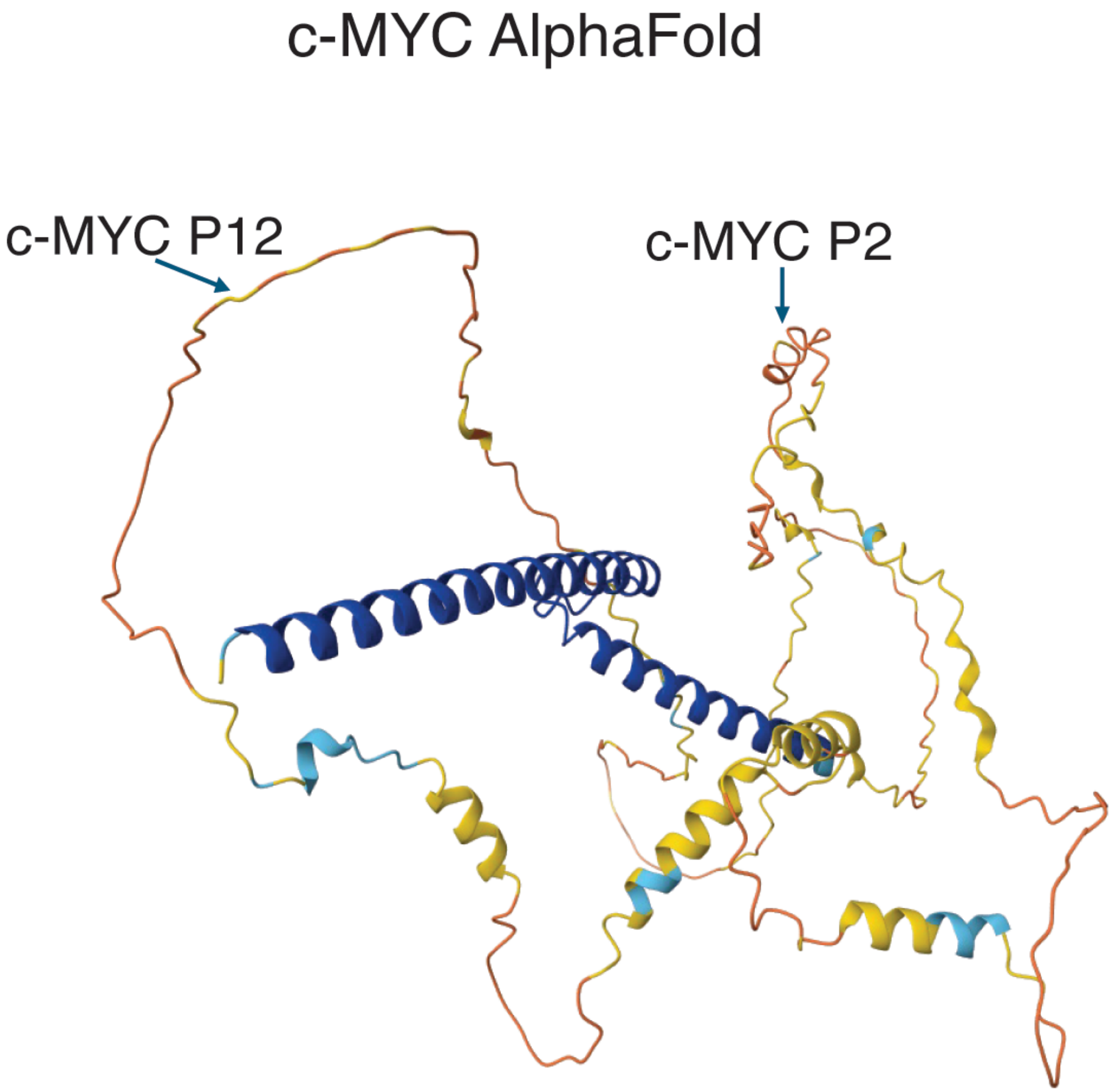

Figure S3: The amyloidogenic P2 and P12 regions are disordered. (A) Predicted 3D structures of human c-MYC proteins by AlphaFold, with the P2 and P12 regions highlighted.

Figure S4

A

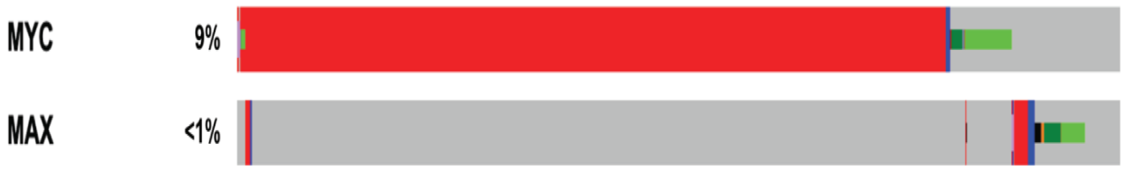

B

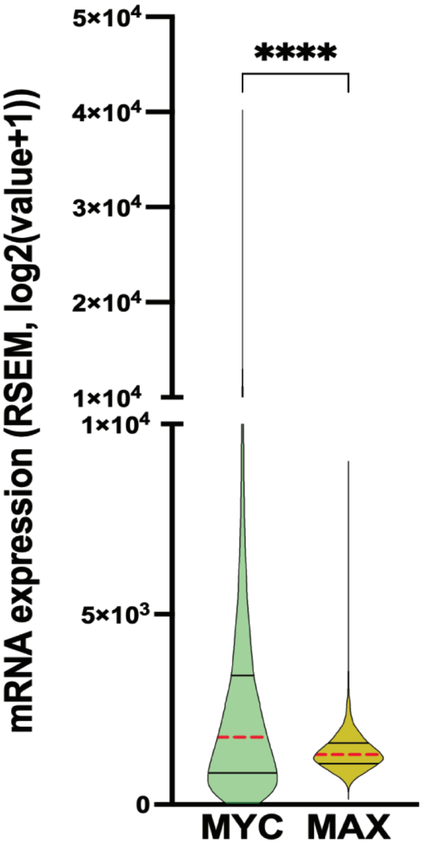

C

Gene	Spearman correlation	*P* value	Pearson correlation	*P* value
MAX	0.06	1.59e-09	0.06	6.17e-10
HSF1	0.22	1.52e-108	0.23	9.09e-125
MXD1	0.16	3.94e-59	0.13	2.33e-36
MXD3	-0.07	3.70e-13	-0.07	2.72e-11
MXI1	0.17	7.70e-69	0.17	2.32e-63
MNT	0.13	1.29e-37	0.11	9.82e-29
MIZ1	-0.09	8.75e-20	-0.07	1.47e-11

Figure S4: The amyloidogenesis of c-MYC contributes to its intrinsic tumor suppressor activity. (A) OncoPrint graph derived from TCGA showing the genomic alterations of *c-MYC* and *MAX* genes in human pan-cancer tissues. (B) Violin plots showing the mRNA expression of *c-MYC* and *MAX* in human tumor samples (median ± IQR, n=10071, Wilcoxon matched-pairs signed rank test). Data are derived from TCGA pan-cancer atlas. RSEM is batched normalized from Illumina HiSeq_RNASeqV2. (C) Gene expression correlation between *c-MYC* and key components of its network in human pan-cancer tissues. Data are derived from TCGA. Genes in red are activators of c-MYC-mediated transcription; genes in black are repressors of c-MYC-mediated transcription.
